# Anisotropic stretch biases the self-organization of actin fibers in multicellular Hydra aggregates

**DOI:** 10.1073/pnas.2423437122

**Published:** 2025-08-04

**Authors:** Anaïs Bailles, Giulia Serafini, Heino Andreas, Christoph Zechner, Carl D. Modes, Pavel Tomancak

**Affiliations:** ^a^Max Planck Institute of Molecular Cell Biology and Genetics, Dresden 01307, Germany; ^b^Center for Systems Biology Dresden, Dresden 01307, Germany; ^c^Cluster of Excellence Physics of Life, Dresden 01307, Germany

**Keywords:** self-organization, nematic order, actin, tissue mechanics, Hydra

## Abstract

During development, groups of cells generate shape by coordinating their mechanical properties through an interplay of self-organization and prepatterning. Hydra displays a striking planar pattern of actin fibers at the organism scale, and mechanics influence the morphogenesis of biological structures during its prepatterned regeneration. However, how mechanics participate in the formation of an ordered pattern from a totally disordered state remains unknown. To study this, we used cellular aggregates formed from dissociated Hydra cells, which initially lose all actin polarity yet regenerate a long-range actin pattern. We showed quantitatively that the actin meshwork evolves from a disordered symmetric state to an ordered state in which rotational symmetry is broken, and translation symmetry is partially broken, with the nematic and smectic order parameters increasing over days. During the first hours, the actin meshwork displayed spatial heterogeneity in the nematic order parameter, and ordered domains separated by line defects progressively grew and fused. This suggests that local cell–cell interactions drive the transition from disorder to order. To understand the mechanism of ordering, we perturbed the tissue’s physical constraints. We showed that while topology and geometry do not have a direct effect, anisotropic stretch biases the emerging orientation of the actin meshwork within hours. Surprisingly, although a Wnt head organizer is expected to play a role in the actin ordering, the stretch-associated alignment happened without the prior formation of a head organizer. This demonstrates the role of tissue mechanics in the alignment of the actin fibers during the disorder-to-order transition.

## Significance

During development, cells generate shape through self-organization and prepatterning. How mechanics impact collective cell organization remains unknown. We used Hydra’s ability to regenerate itself: Aggregates of dissociated cells initially lose actin polarity yet regenerate a striking organism-scale cytoskeleton pattern, with fibers of actin aligned. We showed quantitatively that actin organization evolves from a disordered to an ordered state by growth and fusion of ordered domains. To understand the mechanisms, we perturbed the tissue’s physical constraints. While topology and geometry did not have a direct effect, stretch strongly biased the orientation of actin without the formation of a head organizer. This shows that tissue mechanics can trigger the alignment of actin and the mechanochemical self-organization toward a functional organism.

**Fig. 1.**
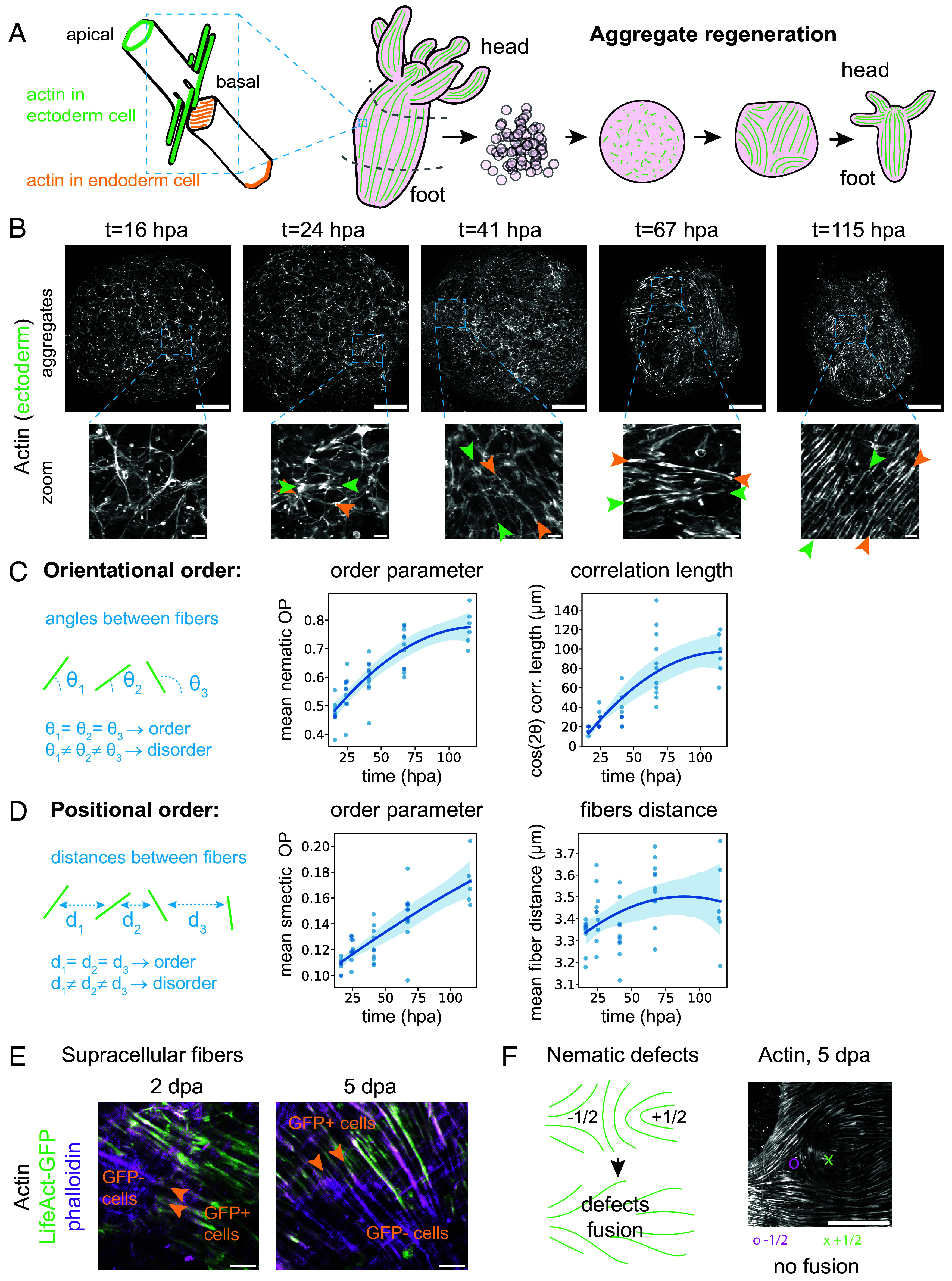
Actin fibers’ orientational and positional order increase with time. (*A*) *Left*, Schematic of actin fibers within an ectodermal and endodermal cell, adapted from ref. 10. *Right*, Schematic of actin fibers during the process of aggregate regeneration. (*B*) Confocal microscopy images of actin in the ectoderm (LifeAct-GFP) of 5 fixed aggregates at 5 time points in aggregate regeneration. Hpa: hours postaggregation. *Bottom*: Zoomed-in images. The green and orange pair of arrows point to the tips of a single actin fiber. Fibers at later time points are longer and more parallel. (*C*) *Left*, Schematic of the concept of orientational order, which measures whether the angles θ between neighboring fibers are similar: The more the angles are similar, the more ordered the fibers are. *Center*: Orientational order parameter (nematic OP) with time. *Right*: Correlation length with time, defined as the distance with a 20% correlation between cos(2θ) values. (*D*) *Left*: Schematic of the concept of positional order, which measures if the distances d between neighboring fibers are similar: The more the distances are similar, the more ordered the fibers are. *Center*: Positional order parameter (smectic OP) with time. *Right*: Characteristic fibers distance. For the 4 plots in (*C*) and (*D*), one dot is the spatial average of one aggregate, with n = 46 aggregates from N = 3 batches, and the blue line is a 2nd-order polynomial regression as a visual guide, with a lighter area representing the CI. (*E*) Confocal microscopy zoomed-in image of actin (phalloidin and LifeAct-GFP) in mosaic aggregates made of a mix of GFP- and GFP+ cells. The arrows show examples of fibers that appear continuous between GFP+ and GFP- cells. (Scale bars 10 μm.) Representative of n = 3 aggregates at 2 dpa and n = 4 aggregates at 5 dpa. (*F*) *Left*, Schematic of expected defect behavior. *Right*, Confocal microscopy zoomed-in image of actin (phalloidin) in a 115 hpa aggregate, showing an example of a pair of −1/2 (purple dot) and +1/2 (green cross) defects. n = 8 WT aggregates from N = 1 batch fixed at 115 hpa displayed 5 pairs of similar defects in total. All microscopy images are maximum-intensity stack projections. Scale bars are 100 μm, except in zoom views, where they are 10 μm.

While self-organization plays an important role in the development and regeneration of animals, it is often coupled with some level of prepatterning or external cues, such as the deposition of maternal RNAs or sperm entry. In vitro cellular systems such as organoids have shown that, in principle, self-organization is sufficient to create cellular patterns (1, 2). However, mechanical forces can influence pattern formation in development (3, 4), and the mechanisms by which cells use mechanics to self-organize in the absence of prepatterning and external stimuli remain unknown. A vintage “organoid,” *Hydra vulgaris*, can form a complete animal from an aggregate of 10,000 to 100,000 dissociated cells within days and without cell proliferation or external inputs (5, 6). Its main body axis is patterned by a gradient of Wnt protein activity and is characterized by a striking actin fibers alignment (Fig. 1*A*). This raises the possibility that the actin meshwork interacts with biochemical patterning during body axis regeneration (7), making it an ideal model system to study the mechanochemical principles of cellular self-organization. Recent biophysical studies in Hydra have focused on the dynamics of topological defects in the actin meshwork and its effect on morphogenesis using regeneration of tissue fragments (8–11) that inherit the actin pattern from the adult axis (8, 12). Here, we leveraged Hydra aggregates made from dissociated cells to erase the initial pattern, enabling the study of the transition from a disordered to an ordered actin meshwork from cells to organism scale.

The adult Hydra has a simple body plan consisting of the oral–aboral axis, with a head surrounded by tentacles at one end and an adhesive foot at the other (13–15). The oral–aboral axis and the head are patterned by genes from the canonical Wnt pathway (16–18). The body column of Hydra is made of two layers of epitheliomuscular stem cells, which give Hydra its regenerative abilities. On the contrary, the head and foot cells are terminally differentiated and cannot regenerate. Throughout the body plan, the ectodermal layer outside and the endodermal layer inside are separated by an extracellular matrix (ECM) consisting mainly of collagen. On the basal side, facing the ECM, both the ectoderm and the endoderm cells have contractile actin fibers (myonemes), which allow Hydra to move by contracting its body column (Fig. 1*A*). In the ectodermal layer, actin fibers are aligned with the oral–aboral axis, while the endodermal ones are orthogonal. The actin fibers in different germ layers also differ by size, with ectoderm fibers extending beyond the cell diameter, while the endoderm ones are thinner and shorter (19, 20).

Aggregates can be made from dissociated cells of the body column, thus lacking the biochemical organizer activities of the head and foot. During the first phase, which lasts around 10 h, endodermal and ectodermal cells spontaneously sort and reestablish the epithelial bilayer and the ECM (21, 22). A lumen appears at the center of the sphere, which then undergoes cycles of inflations followed by rupture of the epithelium and abrupt deflation (23–26). After a few days, protrusions and tentacles appear (Movie S1), followed by the formation of one or several functional heads. The resulting multiheaded Hydra composed of mixtures of cells from many individuals can feed and reproduce, reestablishing organismal identity. The basal actin fibers are lost following the dissociation step (20). Therefore, unlike in Hydra tissue fragments, the actin alignment directional cues are erased in the aggregates. The tissue-scale fiber pattern is later established de novo (20); however, the dynamics and biophysical mechanisms of this process remain unknown.

In this work, we leverage the Hydra aggregates devoid of biochemical and mechanical prepatterns to study how the actin fibers pattern evolves from a disordered to an ordered phase over time, using a combination of imaging, image analysis, and perturbations. We describe the physical parameters governing the transition from disorder to order, investigate the influence of physical constraints on this transition, and demonstrate a direct effect of anisotropic stretch on actin alignment emergence.

## Results

### Actin Fibers’ Positional and Orientational Order Increase with Time.

Using cells expressing LifeAct-GFP in the ectoderm (19) to label actin, Seybold et al. observed that actin fibers are entirely lost during cell aggregate formation, form again at 12 hours postaggregation (hpa), align at the cell scale by 36 hpa, and start to align at the tissue scale at 36-48 hpa (20) (Fig. 1*A*). We build on this initial qualitative description by imaging aggregates fixed at different time points after aggregation across 5 d (at 16, 24, 41, 67, and 115 hpa) and quantifying actin fiber alignment (Fig. 1*B*). The fibers were apparent at the cell scale from 24 hpa and acquired an alignment at the tissue scale from 41 hpa. This alignment improved with time (Fig. 1*B*). We developed an image analysis pipeline to extract relevant quantitative descriptors of the degree of order of the fibers (*Materials and Methods* and *SI Appendix*, Fig. S1*A*). First, we were interested in the loss of orientational symmetry among the fibers (Fig. 1 *C*, *Left*). When the fibers point in random directions, the actin field is isotropic or rotationally symmetric. This symmetry is broken when some directions are favored, initially at the scale of a patch of cells, then at the scale of the entire aggregate. This phenomenon is measured by the nematic order parameter $S=\langle \text{cos}\left(2\theta \right)\rangle$ (*Materials and Methods*), which is 0 for a fully disordered actin pattern and 1 for a fully ordered one. In Hydra aggregates, the nematic order parameter mean increased steadily from 0.47 ± 0.05 at 16 hpa to 0.77 ± 0.06 at 115 hpa (samples mean ± SD, Fig. 1 *C*, *Center* and *SI Appendix*, Fig. S1*B*). The order parameter value at 16 hpa was not 0, even though very few basal actin fibers are present at this stage, which suggested that our quantification captured other image features. However, its increase did not follow the same trend as other measures based on the intensity of the actin meshwork (*SI Appendix*, Fig. S1*C*), showing that the order parameter did not trivially depend on imaging conditions. In addition, the size of the aggregates did not account for the increase, and their average diameter did not change with time (*SI Appendix*, Fig. S1*D*). The scale at which the alignment persists can also be measured by the characteristic length extracted from the autocorrelation function of the fibers’ angles (*Materials and Methods*). Because of the curvature of the tissue, our measurement is an underestimation of the real length. It increased from 17.2 ± 3.6 μm to 94 ± 22 μm over the duration of the experiment (mean ± SD, Fig. 1 *C*, *Right* and *SI Appendix*, Fig. S1*B*). Hence, we provided a quantitative estimate of the global increase in the actin nematic order of Hydra aggregates and showed that this increase occurred progressively over 5 d. The transition from disorder to order appeared smooth, without any noticeable abrupt change.

In addition, we observed that while fibers do not initially have a fixed distance between them, their position, and hence the distance between them, become more regular with time. In other words, the actin field is initially translational symmetric, and this second symmetry may also be broken later when the fibers acquire an array-like pattern along one dimension. This is analogous to the layers formed in the smectic phase of liquid crystals, which have a fixed distance between them. More precisely, the Hydra actin fibers on a 2D surface are analogous to the 2D layers in a 3D liquid crystal volume. Therefore, we devised a second (smectic) order parameter associated with the characteristic distance between parallel fibers. This order parameter measures the positional symmetry breaking and closely matches the smectic parameter used for liquid crystal smectic (27) (*Materials and Methods*, Fig. 1 *D*, *Left*, and *SI Appendix*, Fig. S1*A*). It spans values between 0 and 1. The smectic order parameter increased steadily as the aggregate matured, from 0.108 ± 0.005 to 0.172 ± 0.018 (mean ± SD, Fig. 1*D*). The associated averaged characteristic distance between fibers (or smectic-like layers) remained stable at 3.42 ± 0.15 μm (all time samples mean ± SD), which is compatible with the typical distance between fibers observed in Hydra images, and less than the distance between cell junctions (typically 15 to 30 μm). The increase in the order parameter thus corresponds to the distribution of fiber distances narrowing around this average value. In addition to positional symmetry breaking, smectic liquid crystals possess a second property: They form continuous layers (here, in Hydra, continuous actin fibers). We evaluated the presence of these continuous supracellular fibers by observing actin in WT cells neighboring LifeAct-GFP cells, both stained with phalloidin. The data showed that the fibers are seamlessly continuous from one cell to the next (Fig. 1*E*) in almost all cases at 5 dpa. In some cases, this phenomenon could be observed at 2 dpa already (Fig. 1*E*). Importantly, such a smectic-like organization of the actin fibers in tissues has consequences for the expected evolution of the nematic defects. For example, −1/2 and +1/2 nematic defects close to one another in a nematic field are expected to be attracted and fuse, hence disappearing. However, in a smectic field, in the configuration where layers exist between two defects, they can prevent defect fusion (28). Indeed, we observed examples of −1/2 and +1/2 actin nematic defects close to one another in late WT aggregates when the head and tentacles are already formed (Fig. 1*F*). We would expect such defects to have resolved already, as they do not correspond to biological structures. Although such defect annihilation has been observed in Hydra fragments (10), we speculate that the fibers of adjacent cells are progressively attached by adhesion complexes at the level of desmosome-like junctions (20), which leads to a solid supracellular fiber (or layer) that cannot be easily disassembled. Thus, the layers can prevent defect fusion or slow it down significantly.

Taken together, we show quantitatively that the maturation of aggregated Hydra cells into a functional organism involves a transition of actin fiber orientation and, to a lesser degree, actin fibers position to a higher-order state. Due to the formation of supracellular actin fibers, this ordered state is more akin to a smectic crystal than a nematic one at late stages.

### Spatiotemporal Dynamics of the Nematic Order Increase.

**Fig. 2.**
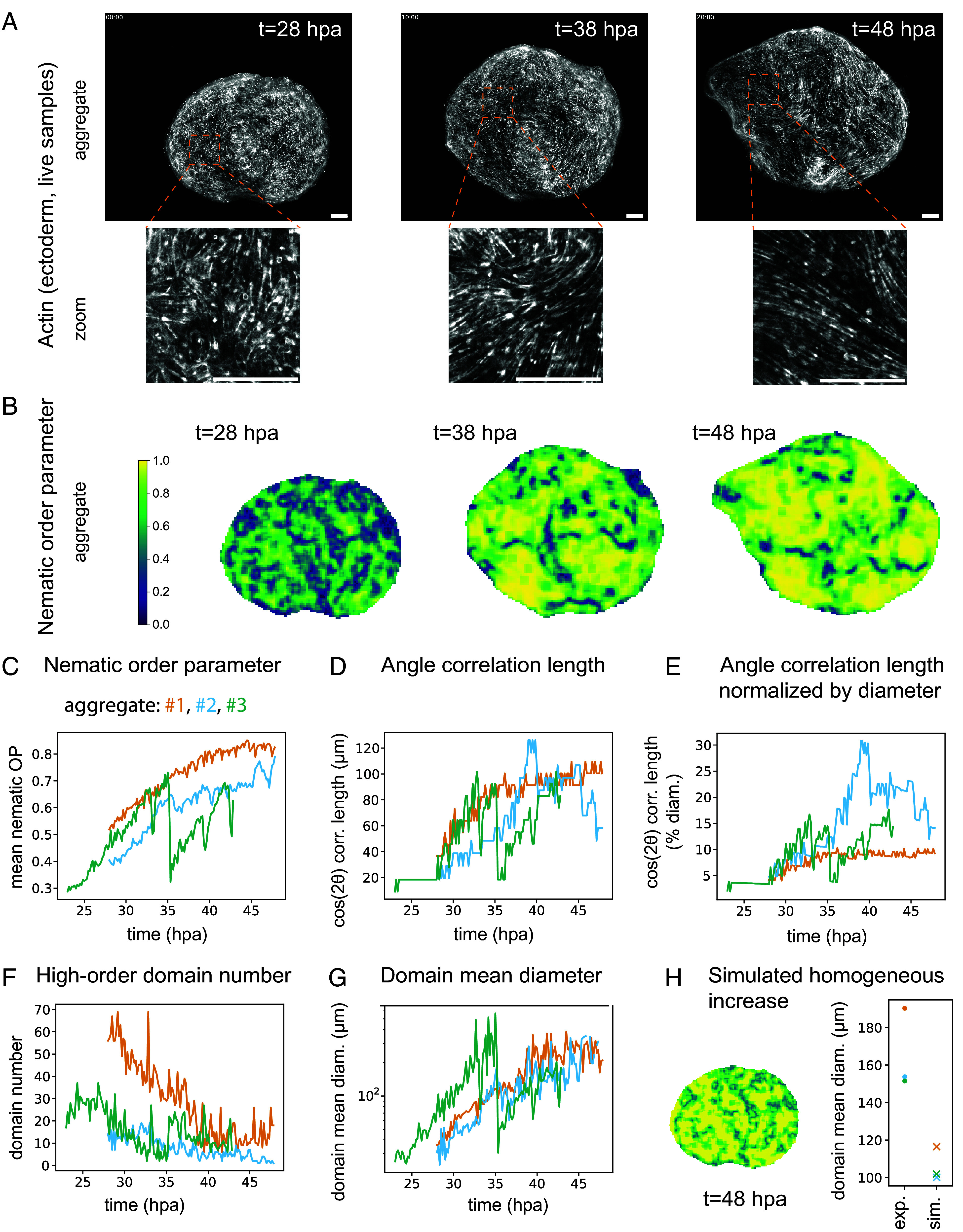
Spatiotemporal dynamics of the nematic order increase. (*A*) Three time points of a light-sheet microscopy movie of actin in the ectoderm (LifeAct-GFP) of a free-floating aggregate starting at 28 hpa. *Bottom*: Zoomed-in images. (*B*) Corresponding spatial measurements of the orientational order parameter (nematic OP). *Left*: Color bar, color code from 0 to 1. (*C*) Spatial average of the nematic order parameter with time of n = 3 live LifeAct-GFP aggregates in water from N = 3 batches. (*D*) Correlation length (defined as in Fig. 1*C*) with time of the same samples. (*E*) Correlation length normalized by the sample’s diameter at each time point. (*F*) High-order domains (defined as in *SI Appendix*, Fig. S2*G*) number with time for the same samples. (*G*) Mean high-order domain diameter with time of the same samples. (*H*) *Left*: Inferred order parameter spatial distribution after 20 h of homogeneous increase starting from the spatial distribution at t = 48 hpa of the aggregate in (*A*). *Right*: Comparison of the mean diameters of high-order domains between real aggregates and their simulated homogeneous growth counterpart. All microscopy images are maximum-intensity stack projections. All scale bars are 100 μm.

In order to resolve the dynamics of actin fibers order increase with a few minutes temporal resolution, we then turned to light-sheet microscopy. We imaged living free-floating and agarose-confined LifeAct-GFP aggregates during the 20 h (from around 26 to 46 hpa) when the actin fibers evolved from cell-scale order to tissue-scale order (Fig. 2*A*, *SI Appendix*, Fig. S2*A*, and Movie S2). During the imaging period, the aggregates underwent one to three inflation cycles (*SI Appendix*, Fig. S2*B*). Outside these global events, we observed some local, seemingly reversible, constrictions of patches of cells (Movie S2). When we quantified the nematic order, the mean of the orientational order parameter increased over one period of inflation, corroborating the observations on fixed data (Fig. 2*C*). The deflation resulted in an instantaneous decrease in the order parameter (compare *SI Appendix*, Fig. S2 *B* and *C*), likely due to the tissue area change and the resulting limitation of our measurement due to imaging resolution (*Materials and Methods*). However, the variation of the order parameter over the long term was not explained by the area change alone (*SI Appendix*, Fig. S2*L*). The angle autocorrelation length also increased, regardless of whether it is measured in microns or as the percentage of the aggregate diameter (Fig. 2 *D* and *E*). Aggregates confined by agarose had, on average, a similar nematic order evolution (*SI Appendix*, Fig. S2 *B*–*F*). The interaggregate variability observed in fixed samples can thus be partly attributed to samples being fixed at different moments in the inflation-deflation cycles, technical variability, and aggregate size variability (*SI Appendix*, Fig. S2*B*).

Interestingly, analysis of the order parameter derived from live imaging of the aggregates in water revealed spatial heterogeneities that evolve with time (Fig. 2*B* and Movie S3). We defined high-order as the areas of the aggregates with an order parameter above a threshold (*SI Appendix*, Fig. S2*G* and *Materials and Methods*) and analyzed the changes of the connected high-order domains over time. The number of such connected domains decreased with time while their area increased (Fig. 2 *F* and *G*). Domain fusion events were observed (Movie S4) and detected in the distributions of individual domain areas (*SI Appendix*, Fig. S2*J*). Eventually, the aggregates comprised only a few ordered domains separated by disordered stripes, also called line defects (Fig. 2*B*). Such line defects would not appear if a concentration gradient was the sole process driving the fiber alignment. However, we cannot exclude that more complex mechanochemical mechanisms based on, e.g., concentration thresholds could produce them. In addition, the domains had higher growth rates and were bigger than they would have been if the order parameter had increased homogeneously (Fig. 2*H*). Such homogeneous increase can happen when there are no local interactions, and instead an external field aligns the system—in Hydra, it could be a chemical field. This suggested that the transition from disordered to ordered nematic field did not occur uniformly across the aggregate but instead by growth and fusion of higher-order domains, likely driven by cell–cell local interactions. The spatial heterogeneities may be initiated or amplified by differences in epithelial tissue state (e.g., epithelial junctions and ECM maturity) and differences in actin fiber maturity, all impacting the tissue mechanics.

We then proceeded to identify what mechanical or physical parameters could facilitate or bias the ordering.

### Topology and Geometry Perturbations of the Actin Meshwork.

**Fig. 3.**
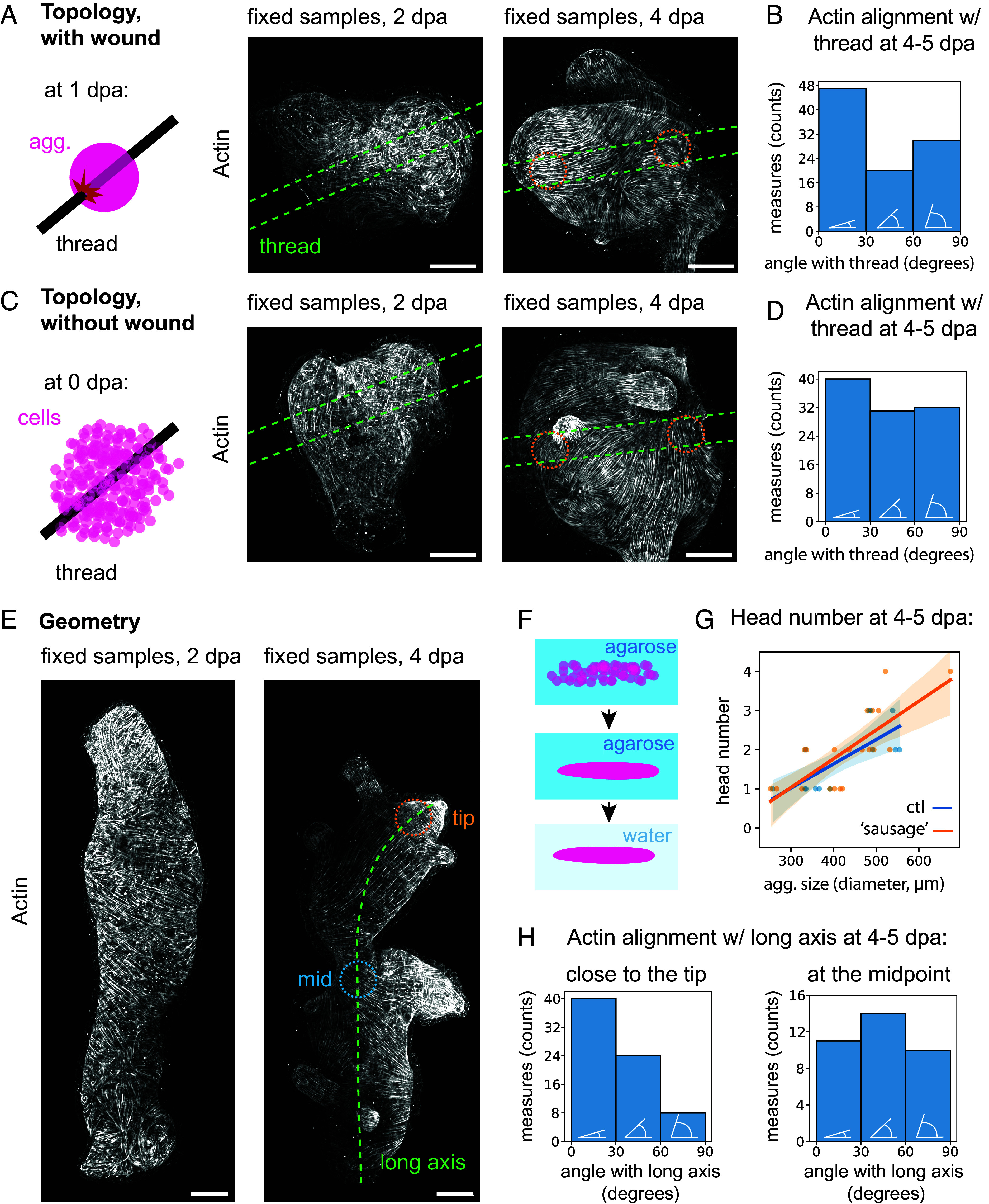
Topology and geometry perturbations of the actin meshwork. (*A*) *Left*, Schematic of the topology perturbation experiment, with tissue wounding. *Right*, Microscopy image of actin (phalloidin) in such a perturbed aggregate fixed at 2 dpa, and another at 4 dpa. The thread contours are the green dashed line drawn from the bright field channel (displayed in *SI Appendix*, Fig. S3). The orange dashed circles show the approximative regions where the manual measurements are performed. (*B*) Manual measurements histogram of the actin angle with respect to the thread, in the regions close to the thread as highlighted in (*A*), at 4 to 5 dpa, binned in parallel (0 to 30°), diagonal (30 to 60°), and orthogonal (60 to 90°) categories, represented with white schematics. 97 measures from n = 25 aggregates, N = 3 batches. (*C*) *Left*, Schematic of the topology perturbation experiment, without tissue wounding. *Right*, Microscopy image of actin (phalloidin) in such a perturbed aggregate fixed at 2 dpa and 4 dpa. The thread contours are the green dashed line drawn from the bright-field channel (displayed in *SI Appendix*, Fig. S3). The orange dashed circles show the approximative regions where the manual measurements are performed. (*D*) Manual measurements histogram of the actin angle with respect to the thread, in the regions close to the two thread ends as highlighted in (*C*), at 4 to 5 dpa, binned in 0 to 30°, 30 to 60°, and 60 to 90° categories. 103 measures from n = 27 aggregates imaged from both sides of the sample, N = 3 batches. (*E*) Microscopy image of actin (phalloidin) in aggregates with a perturbed geometry fixed at 2 dpa and at 4 dpa. Green dashed line: longest curvilinear axis. Orange dashed circle: tip region measured. Blue dotted line: midpoint region measured. (*F*) Schematic of the geometry perturbation. (*G*) Manual count of the number of heads at 4 to 5 dpa in nonperturbed (ctl, n = 13 aggregates from N = 2 batches) and geometrical perturbations (“sausages,” n = 20 aggregates from N = 3 batches) as a function of aggregate size. One dot is one aggregate, and the lines are linear regression as a visual guide, with a lighter area representing the CI. (*H*) Manual measurements histogram of the actin angle with respect to the long axis, in the regions at the tip (*Left* plot) and midpoint (*Right* plot) as highlighted in (*E*), at 4 to 5 dpa, binned in 0 to 30°, 30 to 60°, and 60 to 90° categories. 72 measures (for the tips, imaged from both sides of the sample) or 32 measures (for the midpoint, also from both sides) from n = 18 aggregates, N = 3 batches. All microscopy images are maximum-intensity stack projections. All scale bars are 100 μm.

As the alignment of the ectoderm actin meshwork in Hydra fragments is characterized by the striking formation of a +1 topological defect correlating with the head organizer (10), we sought to perturb the *topology* of the tissue in the aggregates to see whether it has an impact on the nematic field. At first, we created an extrinsic hole by laser puncture of the tissue of ~20 hpa aggregates (when the tissue scale order is not in place yet) and imaged them until the actin meshwork reached a large-scale nematic order. Upon puncture, the aggregate deflated, and rapid wound healing occurred. After that, the normal inflation-deflation cycle restarted. However, the actin meshwork did not develop defects, not even transiently, at the position of the laser puncture (*SI Appendix*, Fig. S3*A* and Movie S5). This shows that a temporary tissue topological defect, such as a hole, does not directly affect the basal actin fiber alignment. To sustain the topological perturbation, we pierced 16 hpa old aggregates with a thread and left the aggregates to develop with the thread inside (Fig. 3*A*, *SI Appendix*, Fig. S3*B*, and Movie S6). This created two permanent topological defects in the tissue corresponding to holes in the epithelial layers (*SI Appendix*, Fig. S3*C* and Movie S7). Previous work used thin wires to generate tissue topological defects in Hydra fragments, with no measurable bias in the position of the head (29). However, fragments conserve an intrinsic tissue planar polarity and alignment of actin, which could be stable to this perturbation, and a bigger thread could have a stronger effect. Hence, we performed similar measurements with aggregates and wider, 50-μm-diameter nylon threads (i.e., of the area of a patch of ~3 to 4 cells). Following the placement of the thread, at 2 days postaggregation (dpa), actin (stained with phalloidin) was still partly disorganized (Fig. 3*A*), and no bias was observed (*SI Appendix*, Fig. S3*D*). Later, at 4 dpa, the actin pattern was well defined and long ranged, and ectoderm fibers were easily distinguishable from endoderm ones by their diameter. At this stage, we manually measured the angle of the ectoderm actin pattern with respect to the thread, in proximity to both holes and on both sides of the samples (Fig. 3*A*). The results showed a significant difference to a uniform distribution (Kolmogorov–Smirnov ks-test, *P* = 0.0074), and parallel alignment was measured in ~50% more cases compared to orthogonal alignment (Fig. 3*B* and *SI Appendix*, Fig. S3*E*). This indicated that the thread was introducing a bias in the actin meshwork. However, this could be caused by the side effects of the piercing process, such as wounding or deformation of the tissue. To uncouple these effects from tissue topology, we used the unique property of the aggregates: By introducing the thread at 0 hpa, when the cells have not built adhesion and the tissue is not formed, topological defects (here holes in the epithelia) can be created by displacing cells without damaging a tissue (Fig. 3*C* and *SI Appendix*, Fig. S3*C*). In this case, we observed a smaller and nonsignificant bias of actin alignment with respect to the thread axis (ks-test *P* = 0.071, ~25% difference between parallel and orthogonal alignment) (Fig. 3*D* and *SI Appendix*, Fig. S3*E*). The head number, axis number, or actin meshwork topological defect number was not limited to two by the two tissue topological defects but rather scaled with aggregate size, with or without wounding (*SI Appendix*, Fig. S5 *A* and *B*). Therefore, persistent tissue defects associated with tissue wounding or deformation slightly bias the actin pattern, while tissue topological defects alone do not significantly bias the actin meshwork.

Another possible source of bias that could orient the actin domains is the overall geometry of the aggregate. Geometry has been shown to orient actin in mammalian epithelial cells cultured on cylinders with radii <40 μm (30) and up to 200 μm (31). To test the effect of *geometry* on actin alignment in Hydra, we created aggregates with a large aspect ratio by centrifugating cells in a 200 μm diameter capillary. We then placed the elongated aggregates (“sausages”) in 2% agarose overnight to prevent their rounding up (Fig. 3*F*). Once the epithelialization of the aggregate was complete and the cells did not sort significantly anymore (>16 hpa) (21), we removed the aggregates from their agarose mold and let them develop in water (Movie S8). This resulted in aggregates in which the geometry had been permanently changed (Fig. 3*E* and *SI Appendix*, Fig. S4 *A* and *B*) as the high aspect ratio persisted over 4 d (*SI Appendix*, Fig. S4*C*). This geometry was now the new preferred state of the tissue and was likely not associated with residual stress. The “sausage” aggregates had a small curvature (a large radius) along the long axis and a high curvature (a short radius) along the short axis (Movie S9). At 2 dpa, the fibers were still not aligned at a large scale (Fig. 3*E* and *SI Appendix*, Fig. S4*A*), making measurements difficult, but the tip of the aggregate seemed to favor parallel actin orientation (*SI Appendix*, Fig. S4*E*). At 4 dpa, the tips of the elongated aggregates displayed a significant bias in ectoderm actin orientation compared to a uniform distribution, with five times more parallel alignment with the long axis compared to the orthogonal one (ks-test *P* = 2.1e-5) (Fig. 3 *E* and *H* and *SI Appendix*, Fig. S4*F*). However, the midpoints did not show such a bias (Fig. 3*H* and *SI Appendix*, Fig. S4*F*), and we observed frequent orthogonal rather than parallel alignment of actin at the narrower part of the tissue (*SI Appendix*, Fig. S4*G*). This showed that the anisotropy in curvature cannot account for the bias in favor of the parallel orientation at the tip, as the difference between the principal curvature values is much higher at the midpoints than at the tip. Having excluded curvature, we analyzed the distribution of +1 actin meshwork topological defects as proxies for the head organizer position across the "sausage" aggregate to understand why actin aligns with the tips. We measured the average half distance between two +1 defects to obtain the average random distance between a point and the closest defect. Next, we measured the distance between tips and the closest +1 defects. Since the result shows that the +1 defects are significantly closer to the tips than other points in the aggregate (Mann–Whitney *U* test *P* = 0.0021) (*SI Appendix*, Fig. S4*D*), the bias in actin orientation measured at the tip reflects the head-organizer attraction to the tip rather than actin orientation in response to curvature. The anisotropic shape and the presence of two tips did not constrain the number of heads, axes, or actin topological defects of the animal, as it scaled similarly to the spherical controls with size (Fig. 3*G*, *SI Appendix*, Fig. S5 *A* and *B*). Yet, the body plan of these aggregates seems to often keep a linear organization (*SI Appendix*, Fig. S5 *C* and *D*). Thus, geometry can bias but does not strictly constrain the axis formation.

### Stretch Aligns the Actin Meshwork without the Prior Formation of a Wnt3 Head Organizer.

**Fig. 4.**
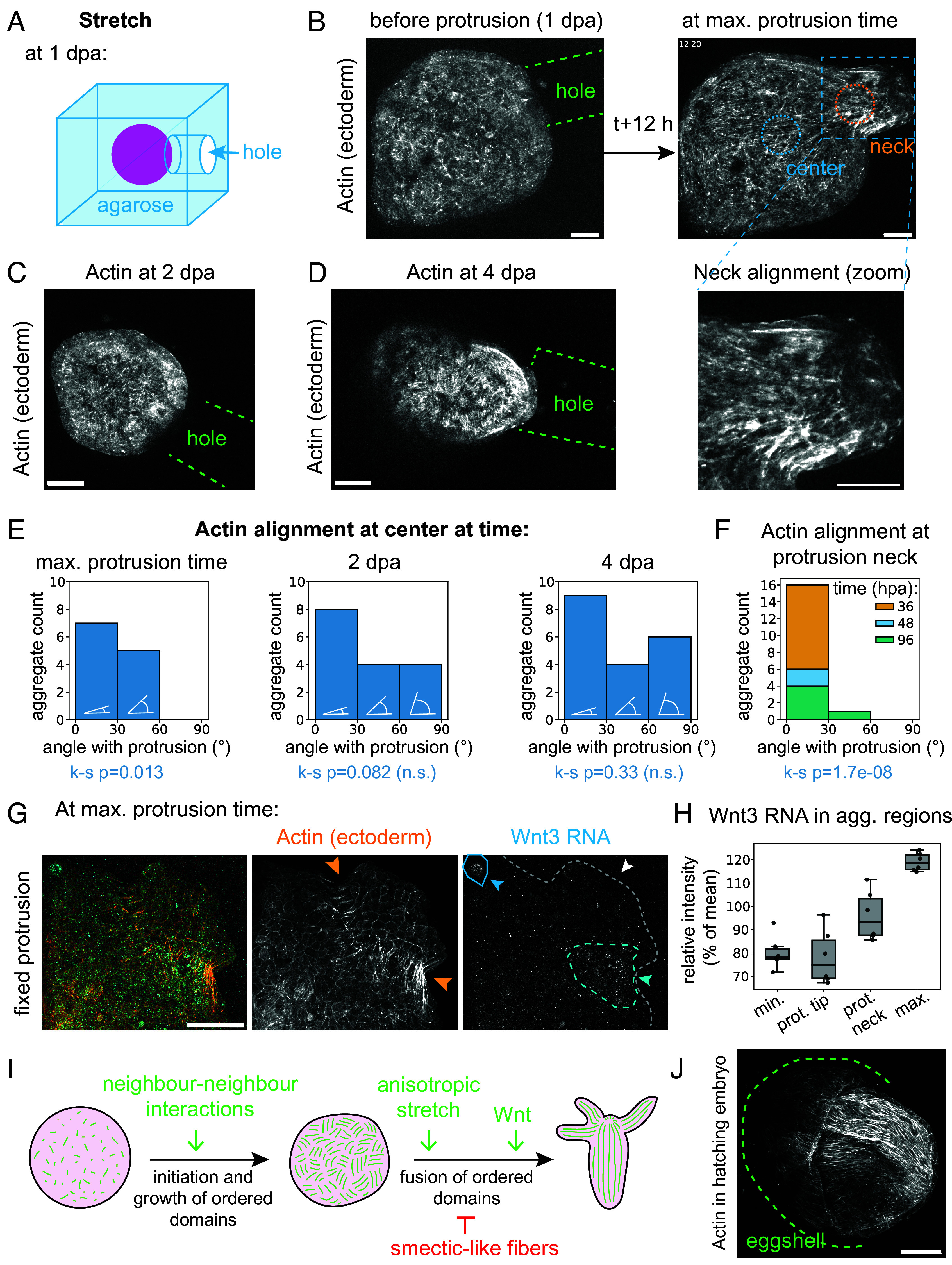
Stretch aligns the actin meshwork. (*A*) Schematic of the stretch perturbation experiment. (*B*) Spinning disk (SD) microscopy images showing actin in the ectoderm (LifeActGFP) in such a perturbed aggregate, before the protrusion (just after the creation of the hole) and after 12 h (around the maximum protrusion time). Green dashed line: hole contours from bright-field images in *SI Appendix*, Fig. S4*B*. Blue circle: example of the center measurement region. Orange circle: example of the neck measurement region. *Bottom Right*: Zoomed-in image of the neck. (*C*) SD microscopy live image of actin in the ectoderm (LifeActGFP) in a 2 dpa (days after aggregation) aggregate placed in the agarose hole at 1 dpa. (*D*) SD microscopy live image of actin in the ectoderm (LifeActGFP) in a 4 dpa aggregate placed in the agarose hole at 1 dpa. (*E*) Manual measurements histogram of the actin angle with respect to the center–hole axis, in the center region as highlighted in (*B*), binned in 0 to 30°, 30 to 60°, and 60 to 90° categories. *Left*: At maximum protrusion time, n = 12 aggregates, N = 4 batches. Center: At 2 dpa, n = 16 aggregates, N = 4 batches. *Right*: At 4 dpa, n = 19 aggregates, N = 4 batches. (*F*) Manual measurements histogram of the actin angle with respect to the protrusion axis, in the neck region as highlighted in (*B*). The measures of the aggregates showing a protrusion at three different time points are stacked. n = 17 protrusions necks, N = 4 batches. (*G*) HCR RNA in situ hybridization with a *Wnt3* antisense probe in two examples of aggregate protrusions fixed 6 h after 1 dpa hole creation. *Left*: Composite image. Center: Ectodermal actin (LifeActGFP). The orange arrows show the neck sides. *Right*: Background-subtracted *Wnt3* channel, with the aggregate contoured in a gray dashed line. The blue arrow and plain line circle show a Wnt3 organizer already present in the sample, but not at the protrusion. The light blue arrow and dashed blue line show a portion of the neck with some *Wnt3* signal. The white arrow shows the protrusion itself, without any signal. (*H*) Normalized *Wnt3* background-subtracted intensity measurements in different regions: the minimal intensity region, the protrusion tip, the protrusion neck, and the maximal intensity region, in n = 6 aggregates from N = 2 batches. (*I*) Proposed model for actin alignment in aggregates. (*J*) Actin (phalloidin) in a hatching Hydra egg. The dashed green line shows the eggshell as per the bright-field image. All microscopy images are maximum-intensity stack projections. All scale bars are 100 μm.

What could attract the head organizer to the tip of the sausage aggregate? We hypothesized that the organizer position is biased due to the tip region experiencing higher stretches during inflation. To test whether deformation affected actin, we proceeded to perturb *stretch* (here, meaning extensile strain) in the tissue specifically. The stretch in a control aggregate is approximately isotropic when the lumen inflates. To create an anisotropic deformation, we placed 1-d-old LifeAct-GFP aggregates in agarose except for a 200-μm diameter cylindrical hole and imaged them live (Fig. 4*A*, *SI Appendix*, Fig. S6*A*, and Movie S10). Our imaging set-up in agarose faithfully captured the basal fibers (*SI Appendix*, Fig. S7 *A*–*D*). During the aggregate osmotic inflation, the agarose restricted the tissue deformation toward the position of the hole (Movie S10 and Fig. 4*B*). This resulted in a local anisotropic stretch in the cells, as evidenced by their strongly anisotropic shapes, with aspect ratios >2, aligned with the protrusion direction (*SI Appendix*, Fig. S6 *B*–*D*). This dramatically affected the actin alignment, with fibers forming almost exclusively in the direction of the hole at the protrusion “neck” (Fig. 4*F*). The effect extended to the aggregate center, where the actin fibers were mainly parallel or diagonal to the hole (ks-test *P* = 0.013) (Fig. 4*E* and *SI Appendix*, Fig. S6*E*). This alignment of the actin fibers was relatively fast, visible as early as 3.1 h, and within 5.3 ± 3.5 h (median ± SD, n = 11, N = 4) after agarose hole formation (Movie S10). The effect persisted over days at the “neck” of the subset of aggregates, showing a lasting protrusion (ks-test *P* = 1.7e-8) (Fig. 4*F* and *SI Appendix*, Fig. S6*F*), but the alignment close to the center vanished progressively (ks-test *P* = 0.082 at 2 dpa and *P* = 0.33 at 4 dpa) (Fig. 4 *C*–*E*). The aggregates were often seen rotating within the agarose confinement after 2 to 3 d (Movie S11), but not before the first deflation as evidenced by movies of aggregates in agarose with labeled cells (Movie S12). Due to this late rotation, we could not conclusively assess whether the orientation of actin alignment persisted over long time scales. However, it was sometimes possible to observe striking and stable actin alignment in the protrusion within the agarose hole, even at 4 to 6 dpa with or without the presence of a head (*SI Appendix*, Fig. S6*F*). Hence, this experiment suggested that the *stretch* of the tissue has a much more profound impact on the actin pattern than tissue *topology* or *geometry*.

We next asked whether the actin alignment induced by mechanical stretch primarily depends on head organizers developing within the regenerating aggregate. In Hydra, it has been established that Wnt signaling aligns actin, as grafting a piece of head on the body column locally changes actin orientation (32). The *Wnt3* expression is increased by osmotically induced stretch (33) and wounding (34), and patches of Wnt expression likely to correspond to head organizers are present early in aggregates (18). To investigate whether the actin alignment upon protrusion formation is due to wound-induced or mechanically induced *Wnt* expression and head organizer formation in the protrusion tip, we imaged LifeAct-GFP aggregates in the local stretch-inducing confinement in agarose with a hole (*SI Appendix*, Fig. S8*A*). We fixed them once a protrusion was prominent (i.e., 5 to 10 h after agarose hole formation) and visualized *Wnt3* expression in these anisotropically stretched aggregates by whole-mount HCR RNA in situ hybridization with a *Wnt3* antisense probe. This probe reliably labels *Wnt3* in the head organizer in adults and regenerated aggregates (*SI Appendix*, Fig. S8 *D*–*G*). Even after the HCR protocol, the protrusions were still visible (Fig. 4*G* and *SI Appendix*, Fig. S8*B*). We found that the protrusion tip had no head organizer, while it was possible to find mature-looking head organizers elsewhere in the same samples (*SI Appendix*, Fig. S8 *C* and *E*). Instead, protrusions had a similar *Wnt3* expression as the regions of the aggregate with the low *Wnt3* intensity. Although the neck showed higher expression, it was not significantly higher than in other parts of the aggregate (Fig. 4*H* and *SI Appendix*, Fig. S8*H*). Hence, while Wnt signaling could still be acting at some level, the aggregate-scale actin alignment is not caused by the prior presence of a head organizer at the tip of the protrusion. This opens the possibility that the induced anisotropic stretch has a direct role in aligning actin.

Taken together, these data support the role of mechanical cues, such as anisotropic stretch, in aligning actin during the self-organized symmetry-breaking process of regenerating Hydra aggregates.

## Discussion

Overall, we quantified the ordering of Hydra aggregate actin fibers on both long and short timescales. Our measurements revealed that orientational order increases smoothly over a long timescale. Although the positional order rises only slightly, it is associated with the formation of stable connected supracellular actin fibers, making it smectic-like (Fig. 1). On shorter time scales, we showed the presence of spatial heterogeneities in nematic order (Fig. 2). We then physically perturbed the aggregates to gain insight into the possible mechanisms of actin fiber alignment. Perturbations of tissue topology and geometry suggested that both have an indirect effect (possibly through wounding or stretch) rather than directly affecting actin alignment (Fig. 3). Conversely, stretch had a strong and fast effect on aligning actin without the previous formation of a head organizer-like structure, as evaluated from *Wnt3* mRNA localization (Fig. 4).

We thus propose a model where the actin fibers self-organize in Hydra aggregates in two steps, possibly overlapping in time (Fig. 4*I*). First, at early stages, the actin fibers interact locally at the cell scale, and these neighbor–neighbor interactions lead to the rotational symmetry breaking in domains of aligned actin that grow over time. Second, inflation of the mechanically heterogeneous tissue would generate anisotropic stretch localized to disordered regions, which helps domain fusion, leading eventually to the long-range ordering of the fibers at the tissue scale. At late stages, the actin orientation would interplay with chemical signaling (in particular, Wnt) to generate +1 actin topological defects (in principle unstable due to their higher energy than a pair of +1/2 (27)), ensuring the match between the tissue genetic patterns (heads, feet, tentacles) and the actin pattern. Both the presence of the anisotropic stretch and the actin interplay with Wnt remain to be characterized.

The exact nature of the local interactions of intracellular actin fibers leading to their alignment has yet to be discovered. It could be steric (with the mere fibers’ presence restricting the orientation of its neighbors), mechanical (with adhesion between fibers or to the ECM), or chemical (for example, Wnt signaling and planar cell polarity (PCP) signaling between neighboring cells). We provided evidence that symmetry breaking does not occur homogeneously but by growth and fusion of ordered domains (Fig. 2), likely amplified by cellular heterogeneities inherent to the aggregate preparation mode. Although the aggregate osmotic inflation provides approximately isotropic stretch, the heterogeneities in local actin order might make some regions more stretch-compliant and lead to localized anisotropic stretch. Since we showed that anisotropic stretch orients the actin fibers robustly and in a long-range (Fig. 4), we hypothesize that this could lead to stable tissue-scale actin orientation in the system. The smectic-like behavior of the actin fibers (Fig. 1) means that the actin meshwork is more ordered than a nematic phase and that another type of defect, *dislocation* defects, must be considered. The smectic-like fibers are likely to prevent the resolution of the line defects between domains. Hence, the role of stretch could be to overcome the associated energy barriers and promote the fusion of domains. The geometry does not influence the actin orientation locally (Fig. 3) but could modify the stretch pattern and the subsequent gene expression (e.g., *Wnt3*) through mechanosensing. The *Wnt3* pattern may emerge in the tissue early on, but the extent of its crosstalk with the actin pattern remains unknown and will require further work. At late stages, the topology inevitably constrains the global number of defects of the actin as a nematic field (with a total charge of +2 on a sphere). When more than two +1 defects are present, this leads to the formation of −1/2 defects, which are not driven by any known chemical pattern or mechanism. However, the topology of the tissue would not have any strong local effect on the actin field (Fig. 3).

Interestingly, the development of aggregates confined by agarose with a hole introduced on one side has some similarities with the process of Hydra egg hatching (35). In the latter case, the embryo, which consists of 2 layers of cells, is constrained by the eggshell until it breaks free from it on one side. A part of the embryo then inflates and stretches outside the eggshell before any morphological signs of a head with tentacles are visible. However, the head of the newly hatched Hydra develops on the side that broke free from the eggshell (35). This suggests the intriguing possibility that aggregate regeneration in the agarose confinement recapitulates mechanisms used during embryonic development. Although hatching Hydra embryos are notoriously difficult to capture, we managed to catch and fix one specimen of Hydra embryo in the act of breaking free from the eggshell. Phalloidin staining revealed that actin is indeed aligned parallel to the protrusion in a hatching Hydra egg stretching half outside of its eggshell (Fig. 4*J* and *SI Appendix*, Fig. S6*G*), while no +1 defects or other typical Wnt-related structures were present. Since Hydras never experience dissociation and reaggregation as part of their normal life cycle, the remarkable reformation capacity of the system may be due to artificially arriving at a state that resembles a physiological embryonic process. An exciting hypothesis is that the anisotropic stretch-induced actin alignment we uncovered participates in the embryo body axis formation or ensures its robustness.

Regardless of the developmental coincidences that make Hydra resilient to dissociation, the ordering of actin fibers in aggregates arises from a complex interplay of chemical, mechanical, geometrical, and topological self-organized constraints. The respective temporal order and causal role of chemical, mechanical, or topological cues are still unclear in Hydra tissue fragments (9, 11, 12, 32), and in principle, the known positive autoregulation of *Wnt3* (36) could be sufficient to create patterns from initial spatial fluctuations. Wnt signaling is a conserved mechanosensing pathway (37) and could be at the origin of the mechanical effects on head formation observed in fragments, as experimental manipulations indicate (9, 33, 38). Theoretical work also emphasized the importance of the coupling between nematic alignment and concentration gradient (39) or the role of stretch-based mechanical feedback in Wnt signaling (9, 38, 40, 41). Aggregates allow the disentangling of these respective contributions by monitoring the de novo emergence of both the Wnt concentration field and the actin field. We showed that actin pattern formation can be uncoupled from the presence of a mature Wnt3 head organizer and provided evidence for a mechanism where the *anisotropy* in stretch instructs the emergence of actin order and axial alignment. In the future, it will be important to assess whether this mechanism has long-lasting consequences on the self-organization of the head organizer in the aggregates, as it seems to be the case in the prepatterned Hydra fragments (38). The interplay and feedback mechanisms between the formation of patterns of Wnt signaling and actin warrant further studies in both Hydra fragments and aggregates.

Beyond Hydra, cells grown in in vitro monolayers align their long axes akin to nematic liquid crystals (42), and nematic topological defects have been associated with tissue rupture or cell extrusion (43, 44). The molecular aspects of the interplay between actin fibers’ orientation, tissue anisotropic stretch or stress, and gene patterning are investigated in systems with powerful molecular toolkits. The cytoskeleton and associated proteins at focal adhesions are inherently mechanosensitive (45–48), and actin fibers formed, modified, or oriented by stress are present in other systems: ventral stress fibers in mammalian migrating cells (49, 50), actin bundles in the development of mammalian striated muscles (51, 52) or smooth muscles (53), and apical stress fibers in insect epithelial tissues (54). It will be interesting to investigate whether what we uncovered also extends to these systems, particularly if they form a smectic phase that prevents the fusion of aligned domains.

Overall, we described the physical parameters governing the transition from disorder to order in a mechanochemical self-organization process and investigated the influences of physical constraints on this transition. While mechanochemical symmetry breaking also occurs in vitro, Hydra aggregate cellular polarity and actin fibers self-organize across scales from heterogeneous mixtures of cells to entire functioning and reproducing organisms, allowing to connect in vitro findings with developmental principles.

## Materials and Methods

### Summary.

*H. vulgaris* polyps were cultured according to standard protocols (20), and aggregates were made of dissociated cells from Wild-Type or LifeAct-GFP lines (19). The aggregates were imaged live with 10× objectives with light-sheet (Fig. 2) or spinning-disk microscopes (Fig. 4) or fixed and imaged with 25× objectives and scanning confocal microscopes (Figs. 1 and 3). Samples were mounted free-floating in FEP cuvettes for light-sheet imaging. Starting with the ImageJ plugin OrientationJ (55), a custom image analysis pipeline in Python was created to measure the nematic and smectic order parameters and the characteristic length. Hybridization Chain Reaction RNA in situ against *HyWnt3* was performed according to published protocols (56, 57), and antisense probes specific for the Hydra *Wnt3* mRNA or GFP mRNA were designed using the software from (58). The aggregate preparation, perturbation, and statistics are detailed below. Extended Materials and Methods on protocols, imaging, and image analysis can be found in *SI Appendix*.

### Aggregate Preparation.

To prepare aggregates, 50 Hydras fed 48 to 72 h earlier are selected. We used WT adults, LifeAct-GFP adults, or a mix of both to create mosaic aggregates (Fig. 1*E*). The body column parts are dissected by cutting below the tentacles and above the bud zone. The following steps are performed at 18 °C using a custom-made Peltier tube cooler. The body columns are incubated in Dissociation Medium, DM (3.6 mM KCL, 6 mM CaCl2, 1.2 mM MgSO4, 6 mM NaCitrat, 6 mM NaPyruvat, 4 mM Glucose, 12.5 mM TES, 50 mg/L Kanamycin, and 100 mg/L Streptomycin, pH 6.9) during 20 min. The DM is removed, 1.5 mL of new DM is added, and then the body columns are gently pipetted up and down with a flamed glass pipette for 30 s, the solution is left to sediment for 2 min, and the supernatant containing single cells and small cell clusters is collected. The cycle of the pipetting-supernatant collection is repeated around 8 times until the pieces of tissue are fully dissociated. The resulting cell suspension is centrifuged at 150 g during 5 min at 4 °C, the supernatant discarded, and the cell pellet resuspended in 4 mL DM, aliquoted in Eppendorf tubes, and centrifuged again at 150 g during 5 min at 4 °C. The tubes are placed face down in a Petri dish containing DM until the cell pellets detach and sink into the Petri dish. The pellets are cut in two, resulting in a ratio of 2 to 3 Hydra body columns used per aggregate. After 2 h, the aggregates are transferred into a 1:1 HM-DM mix, in which they stay overnight. The next day, they are transferred into a 3:1 HM-DM mix, then around 6 h later into pure HM. For 2 out of 3 samples used in Fig. 2, full Hydras were used instead of body columns, and the cell suspension was filtered using a 100 μm diameter sieve before the first centrifugation, making sure to remove big clusters (epithelial cells are 20 to 30 μm in diameter), and the aggregate development appeared similar.

### Perturbations of Aggregate Topology, Geometry, and Deformation.

“Skewer” aggregates (Fig. 3): The aggregates are made as described and pierced using a 50-μm diameter nylon thread with the aid of tweezers, either immediately after being made or the day after. Changes of medium proceed normally, but the medium is removed instead of the aggregates being transferred to a new dish.

“Sausage” aggregates (Fig. 3): The cell suspension is prepared as described until the end of the first centrifugation. The supernatant is then discarded, but no DM is added. The cell suspension is transferred to 200 μm inner diameter glass capillaries, and the capillaries are plugged with dentist paste (Picodent Twinsil) and then centrifuged at 150 g for 5 min at 4 °C. The capillaries are kept for 1 h at 18 °C, and then the elongated pellets (“sausages”) are pushed out of the capillaries into a DM-filled Petri dish. After 1 h, the sausages are transferred into 1:1 HM-DM. 2 h later, they are embedded into 2% low-melting agarose drops, covered with 1:1 HM-DM, where they stay overnight. The next day, the solution is replaced with 3:1 HM-DM. At around 20 hpa, the agarose shell is broken using needles, and the freed aggregates are transferred into pure HM.

“Boxed” aggregates (Fig. 4): The aggregates are prepared as described. The next day, at around 24 hpa, they are embedded in 1 or 2% low-melting agarose, in direct contact with the tip of a 200 μm diameter nylon thread. After the agarose solidifies, the thread is gently pulled away, exerting some force on the aggregate and leaving a cylindrical hole of 200 μm in diameter in the agarose surrounding the aggregate.

### Statistics.

To assess the randomness of actin orientation upon perturbations (Figs. 3 and 4), the measurement distribution was normalized in the range 0 to 1 and tested against a uniform distribution with a Kolmogorov–Smirnov test using the Python function scipy.stats.kstest. All the measurement points per sample were used assuming they are independent, as they are >200 μm from each other, the distance over which correlation in angle orientation disappears (Fig. 1). To assess the difference between the distributions in Fig. 3*H*, we performed a Mann–Whitney test using the function scipy.stats.mannwhitneyu.

## Supplementary Material

Appendix 01 (PDF)

Movie S1. Example of a WT aggregate regeneration in water over days, bright-field imaging. Scalebar: 100 microns. Timestamps: hours after aggregation (hpa).

Movie S2. Actin-tagged (LifeAct-GFP) aggregate regeneration in water from 28 hpa to 48 hpa, mounted in an FEP cuvette and imaged with a light-sheet microscope. Scalebar: 100 microns. Timestamps: hours after aggregation (hpa).

Movie S3. Nematic order parameter value calculated from the aggregate in Movie S2, superimposed on the actin Movie S2. X and Y axis: position in pixels. Colormap: nematic order parameter value. Timestamps: hours after aggregation (hpa).

Movie S4. Zoomed-in view of a fusion event in Actin-tagged (LifeAct-GFP) aggregate regeneration in agarose. The orange arrows show two domains with orthogonal orientation initially that later end up parallel to each other. Scalebar: 10 microns. Timestamps: hours after aggregation (hpa).

Movie S5. LifeAct-GFP aggregate regeneration after laser puncture at 21 hpa, mounted in 1% agarose and imaged with a spinning-disk microscope. Scalebar: 100 microns. Scalebar: 100 microns. Timestamps: hours after aggregation (hpa).

Movie S6. Bright-field movie of the regeneration of an aggregate pierced by a thread at 1 dpa, free-floating in water. Scalebar: 500 microns. Timestamps: hours after aggregation (hpa).

Movie S7. Full z-stack of a phalloidin staining of a 4 dpa aggregate pierced by a thread at 1 dpa, labeled with depth in microns. Scalebar: 100 microns.

Movie S8. Bright-field movie of the regeneration of a ‘sausage’ aggregate, free-floating in water. Scalebar: 500 microns. Timestamps: hours after aggregation (hpa).

Movie S9. Full z-stack of a phalloidin staining of a 4 dpa ‘sausage’ aggregate, labeled with depth in microns. Scalebar: 100 microns.

Movie S10. LifeAct-GFP aggregate regeneration in a 2% agarose box with a hole, imaged just after hole creation, from 22 hpa to 45 hpa, with a spinning-disk microscope. Scalebar: 100 microns. Timestamps: hours after aggregation (hpa).

Movie S11. Later LifeAct-GFP aggregates regeneration in an agarose box with a hole (after being imaged as in Movie S5) from 45 hpa to 88 hpa, bright-field imaging. Scalebar: 100 microns. Timestamps: hours after aggregation (hpa). The aggregates can move inside the agarose (left aggregate) or stay relatively still (right aggregate).

Movie S12. Aggregate inflation in agarose, with cells labeled with a lipid dye. Scalebar: 100 microns. Timestamps: hours after aggregation.

## Data Availability

Microscopy data used in this article as well as the custom codes developed for the nematic and smectic analysis of images are available on a Zenodo repository (59, 60).
