# Supplementary material for: Anisotropic stretch biases the self-organization of actin fibers in multicellular Hydra aggregates
Source: Proc Natl Acad Sci U S A. 2025 Aug 4;122(32):e2423437122. doi: 10.1073/pnas.2423437122 (PMC12358849; doi:10.1073/pnas.2423437122)
Supplement: Supplementary file 1 — Appendix 01 (PDF) [file pnas.2423437122.sapp.pdf]

PNAS

Supporting Information for
Anisotropic stretch biases the self-organization of actin fibers in multicellular Hydra aggregates

Anaïs Bailles[1]*, Giulia Serafini[1], Heino Andreas[1], Christoph Zechner[1,2,3], Carl D. Modes[1,2,3], Pavel Tomancak[1,2,3]*

*Corresponding authors: Bailles, Tomancak
Email: bailles@mpi-cbg.de, tomancak@mpi-cbg.de

This PDF file includes:

Supplementary Methods
Figures S1 to S8
Legends for Movies S1 to S12
SI References

Other supporting materials for this manuscript include the following:

Movies S1 to S12

Supplementary Methods

Hydra culture

Hydra vulgaris lines are cultured according to standard protocols (*1*) at 17± 1°C in Hydra Medium, HM (1 mM Tris, 1 mM CaCl2, 0.4 mM MgCl2, 0.4 mM KCL, 4 mM NaHCO3, pH=7.3-7.6) and fed once or twice a week with artemia nauplii less than 48 hours old. The lines used are the *Hydra magnipapillata* 105 as WT and the *Hydra vulgaris* actin1-Lifeact-EGFP line inserted in the ectoderm (*2*), both gifts from the Hobmayer lab.

Fixation and staining

Fixation: The aggregates are fixed using fresh 4% PFA in HM at room temperature for 45 min. They are rinsed twice in HM, washed for 5 min in HM, washed for 5 min in PBS, and stored at 4°C in PBS. For HCR *in situ*, the PBS wash is replaced by a 1:1 MeOH-HM 5 min wash and a 100% MeOH 5min wash, and the samples are stored at -20°C in MeOH.

Phalloidin staining: After fixation, the samples are incubated for 1h30 in PBST (PBS + 0.05% Triton X-100) at room temperature. Then, they are incubated in PBST with DAPI (1:250) and rhodamine-phalloidin (1:250) at 4°C overnight on a rocking platform. The next day, they are rinsed twice with PBS, washed 2x5 min in PBS, soaked in glycerol (80%)-DABCO overnight, mounted in between two coverslips with tape as spacers, and imaged within a few days from both sides.

Whole-mount HCR RNA *in situ* hybridization

Hybridization Chain Reaction RNA *in situ* against *HyWnt3* was performed according to published protocols (*3*, *4*) with the following modifications: the protocol always started from fixed aggregates conserved in 100% MeOH at -20°C for at least 2-3 days. After the rehydration steps, the samples were incubated for 5 min at room temperature in 1 mL of 10 µg/mL Protease K solution in PBS-Tween 20 (PBT) 0.1%. After the post-fixation step in 4% PFA in PBT 0.1%, the samples were washed 5 times for 5 min in 1 mL PBT 0.1% and once for 5 min in 500 µL 2x SSC-Tween 20 0.1%. The pre-hybridization took place in a volume of 400 µL of probe hybridization buffer, while the O/N incubation with the RNA probes took place in a total volume of 200 µL. Antisense probes specific for the Hydra *Wnt3* mRNA or GFP mRNA were designed using the software from (*5*). GFP probes are used as a negative control (Fig. S8G), since WT Hydras do not have the GFP gene.

Fixed imaging

Fluorescently labeled fixed samples (Fig. 1,3, and 4G) were imaged using a Zeiss LSM 700 microscope equipped with a Zeiss Axio Observer.Z1 inverted stand and a 25x (NA=0.8) multi-immersion objective in glycerol or water depending on the sample, and operated with Zeiss ZEN 2012 SP5 FP3 (black).

LifeAct-GFP (Fig. 1): LifeAct-GFP fixed aggregates were imaged with a 488 nm DPSS laser, a zoom factor of 0.5x, a pixel size of 0.5 microns, and a stack covering from the surface until the equator of the aggregate (around 50 to 100 microns thick), with a z plane spacing of 1.49 microns. Glycerol solution was used for 2 of the batches and water for one of the batches, without any strong visible difference.

Phalloidin+BF+Dapi (Fig. 3): WT fixed aggregates were imaged in glycerol using a laser diode 405 nm (for DAPI), a laser DPSS 555 nm (for rhodamine phalloidin), and a laser DPSS 639 nm (for bright-field), a zoom factor of 0.5x, a pixel size of 0.5 to 1.0 microns and a stack covering from the surface until the equator of the aggregate (around 50 to 100 microns thick), with a z-plane spacing of 1.31 microns.

HCR in situs (Fig. 4): LifeAct-GFP fixed aggregates were imaged in glycerol with a 488 nm DPSS laser (for LifeAct-GFP), a laser DPSS 639 nm (for HyWnt3-Alexa647nm). A zoom factor of 0.5x, a

pixel size of 0.57 microns, and a stack covering from the surface until the equator of the aggregate (around 40 to 80 microns thick), with a z-plane spacing of 2.0 microns were used.

2-Photon imaging (Fig. S7): As a control, we imaged aggregates embedded in agarose using 2-Photon (2-P) confocal microscopy. With the high resolution of a confocal microscope and its high penetration depth, we consider 2-P images to be the 'ground truth' of the presence of actin fibers, yet it is too slow to use on live samples. We used a Zeiss LSM 780 NLO 2-Photon inverted microscope operated by Zeiss ZEN Black version 14.024.201, a 25x (NA=0.8) water immersion objective, a laser multiphoton Coherent Chameleon Vision II 700-1064 nm, and a z-plane spacing of 1.03 um.

Live imaging

SPIM (Fig. 2): Samples were imaged using a Zeiss Light-sheet Z.1 with Zeiss 10x (0.2 NA) Air illumination objectives and a Zeiss Plan Apochromat 10x (0.5 NA) water immersion detection objective, operated with Zeiss ZEN 2014 SP1. Samples free-floating in HM were mounted in custom cubic cuvettes made of 0.05 mm-thick FEP foil, with a ~1.5x1.5 mm^2 section, and attached to a glass capillary. Samples in agarose were mounted using glass capillaries (2 mm outer diameter, ~1.5 mm inner) filled with 1% low-melting agarose according to standard protocols. Both were imaged using a 0.5-1x zoom factor according to the sample size (which results in a pixel size from 0.46 to 0.91 microns), filling up the chamber with Hydra Medium (HM) kept at 18°C. Four-angles view stacks were acquired by exciting the EGFP with a 488 nm DPSS laser at low power, with 30 ms acquisition time per frame and one plane every 6 microns, covering around 540 microns (depending on the sample size). One time point every 10 min was acquired to minimize laser exposure of the sample.

Spinning disk (Fig. S2, Fig. 4): Samples were imaged using a Zeiss Spinning Disk equipped with a Yokogawa CSU-X1 scan head (5000 rpm, pinhole diameter 50 μm, pinhole spacing 250 μm) and a Zeiss AxioCam 705 Mono CCD camera, and a Zeiss Plan Apochromat 10x (0.45 NA) Air objective. The samples were mounted in a glass-bottom MatTek 35 mm dish, covered with 1 or 2% agarose and HM, and kept at 18°C using a Warner cooling chamber. LifeAct-EGFP was excited with a laser DPSS 488 nm 200 ms per frame. A pixel size of 0.69 microns and a z-step of 3.0 microns were used, covering around 300 microns in depth. One time point every 10 min was acquired in order to minimize laser exposure of the sample.

Laser ablation: In combination with the spinning disk system described above, a RAPP optoelectronic 355nm laser (1 kHz rep rate, max 42 μJ pulse power, 1 ns pulse length) operated by Syscon2 was used to cut out a disk in the epithelium. The disk cutting itself lasts ~100-150 ms in total, and we stream recorded (as fast as possible) during the ablation, with a 100 ms exposure time. Long movies were then acquired as above with a 10-minute time step.

Image analysis pipeline

The goal of this pipeline is to extract the coarse-grained actin orientation and measure order parameters, correlation lengths, and high-order domain properties from maximum intensity projections of the stacks of actin signal. The initial angle measurement is done using Fiji and OrientationJ (*6*), while the coarse-graining and further measurements are made with custom Python codes. For fixed images, both sides of the aggregates were analyzed and averaged (Fig. 1). For live imaging (Fig. 2), one side of each aggregate was chosen.

Angles measurement: The OrientationJ 'OrientationJAnalysis' plugin (*6*) was used to obtain the dominant angle of the image features. Briefly, this plugin calculates the image structure tensor from the gradient of the intensity within a window and its eigenvectors to obtain the Coherency and Angle, indicating resp. the magnitude and principal direction of the image gradient. A window with $\sigma = 2$ pixels was used to match the typical width of the actin fibers.

Mask of the aggregate (region of interest): For live samples (Fig. 2), a mask is created in Fiji using a Triangle threshold segmentation of the actin movie, followed by erosion, dilation, and filling hole binary operations. For fixed samples (Fig. 1), the mask is created using Python, first applying a Gaussian blur of sigma=3 to the actin image, then segmenting the blurred image with a threshold of 200 (chosen after manual inspection of the mask obtained for the different samples), followed by 10 iterations of an erosion binary operation done with 20x20 matrices of one's element. The goal of this erosion is to remove the edges of the sample, which do not show the basal actin fibers. The equivalent diameter of the aggregates is obtained by summing the pixels of the mask S and taking $D = 2\sqrt{S}/\sqrt{\pi}$.

Coarse-graining of angles values: To extract the average actin fibers angle at a cellular (rather than at a single fiber-) scale, we coarse-grained the Angle matrix obtained by OrientationJ in boxes of 10x10 microns^2. We implemented a nematic version in Python by calculating the local director angle of each box according to $\theta_{box} = \frac{1}{2}\arctan\left(\frac{<\sin(2\varphi)>_{box})}{<\cos(2\varphi)>_{box}}\right)$, where φ is the angle measured at a 2-pixels scale by OrientationJ, the average is performed over the box region, and the numpy.arctan2 function was used.

Actin signal measurements: Actin intensities are coarse-grained by simply downscaling the raw image, and the average of the average in the mask area is calculated.

Correlation length: From the coarse-grained angle field θ(x,y), and since we are interested in the nematic properties, we calculated C(x,y), the autocorrelation matrix of cos(2θ) using the ITK library in Python and its method MaskedFFTNormalizedCorrelationImageFilter. Note that this method performs the autocorrelation on the masked image, which is important for the correlation length to fall to zero at a length larger than the object size. We then converted it to polar coordinate as C(r, θ) and averaged it radially (i.e., over θ) to obtain the autocorrelation function F(r), with r the distance between points. The correlation length was extracted by either fitting a decreasing exponential to the initial portion of the curve (over 6 microns), since the curve is only exponentially decreasing initially (Fig. S2d) or setting a threshold of 0.2 (i.e., 20% correlation) as the value of correlation corresponding to the characteristic correlation length. Both gave qualitatively similar results (increasing with time), but here we report the threshold of 0.2 length, which better reflects the non-zero correlation remaining at larger distances.

Nematic order parameter: The nematic order parameter was calculated as $S =< \cos(2(\theta - \hat{\theta}) >_{neighb}$, where $\hat{\theta}$ is the neighborhood director calculated as $\hat{\theta} = \frac{1}{2}\arctan\left(\frac{<\sin(2\theta)>_{neighb}}{<\cos(2\theta)>_{neighb}}\right)$. The neighborhood is chosen to be 50x50 microns^2, corresponding to a cell and its immediate neighbors. Deflation events caused a sudden increase in the packing of the fibers, such that their orientations are not detected properly and the nematic order parameter show a sudden decrease that could be artefactual (Fig. S2B,C). However, the order parameter did not correlate locally with the actin fibers' meshwork intensity (Fig. S2K), showing that it did not trivially depend on our imaging. Overall, the order parameter allows us to quantify the order parameter and compare it over long period of time (hours), but we cannot compare on time frame with the time immediately before or after (minutes).

Smectic order parameter: The smectic order parameter would, in principle, be the amplitude of the principal mode of a Fourier decomposition of the actin density. To adapt it to our biological data, we devised a related smectic order parameter, easier to measure, as follows. For each box of 10x10 microns^2, we use the corresponding angle value to select a 10 microns x 50 microns image stripe with its long axis orthogonal to the actin fibers. This becomes a 1D intensity profile I(r) by integrating over the smaller dimension (the one parallel to the nematic orientation). We found 10 microns an appropriate averaging length, as it allows to 'erase' potential discontinuities in the fibers due to either imaging noise or physical discontinuities, without blurring the signal. In practice, for smaller values, the measurement is quite insensitive to the choice of this scale. We derivate it to remove contributions from large-scale intensity variations, then calculate dI/dr autocorrelation function with numpy.correlate, normalize it to 1, and the height of the first autocorrelation peak after

the r=0 peak is defined as the order parameter, while its position gives the characteristic spacing between the fibers.

High-order domains analysis: The threshold for 'High-order domain' is defined by the 10% of highest nematic order parameter values cut-off at time 0. The order parameter images at different time points are thresholded accordingly and the connected domains are identified using skimage.measure.label. This provides the number of domains with time. Their area is measured by counting the number of pixels of a given connected domain.

Homogeneous growth scenario: To provide the equivalent hypothetical homogeneous increase scenario, the spatially averaged order parameter time evolution is smoothed with a uniform filter of size 5, and differentiated to obtain the average rate of increase $\frac{d\bar{S}(t)}{dt} \equiv \frac{1}{N}\sum_i^N \frac{dS_i(t)}{dt}$ (where the sum is over the boxes i). In our homogeneous increase scenario, for a given time t we chose all the boxes to have the same rate of increase $\frac{dS'}{dt}$ except the n_1 boxes which have already reached $1 - \frac{d\bar{S}(t)}{dt}$, such that $\frac{dS'(t)}{dt} = \frac{N}{N-n_1(t)} \frac{d\bar{S}(t)}{dt}$. The order parameter evolution for the boxes is obtained iteratively with:

$$\begin{cases} S_i(t+1) = S_i(t) + \Delta t \frac{dS'}{dt} \text{ if the box evolves} \\ S_i(t+1) = S_i(t) \text{ else} \end{cases}.$$

The synthetic images obtained are then analyzed as described above, and the area values are corrected by multiplying by $A(t)/A(t=0)$, where A is the total area of the sample, to take into account the effect of aggregate inflation.

<u>Manual analysis of actin angle</u>

For perturbation conditions, the ectoderm actin orientation is measured manually within some ROIs using the 'angle' tool from Fiji.

'skewer' aggregates (Fig. 3): The ROIs are chosen to be well-aligned actin zones in proximity (~50 microns in the direction of the other thread tip) to where the nylon thread enters the aggregate (as seen in bright-field images). For one aggregate, 2 such ROIs per side are measured, resulting in 4 measurements per aggregate. The angle is measured as the angle with the nylon thread, 0° being parallel to it, and 90° orthogonal to it.

'sausage' aggregates (Fig. 3): As the sausages are often bent at 4 dpa, the longest curvilinear axis is first defined manually using the 'segmented line' Fiji tool, and its length is measured. The ROIs angles are measured with 0° being parallel to the longest curvilinear axis and 90° orthogonal to it. A total of four measurements are made at the tips of each aggregate (2 sides x 2 tips). The midpoint ROI is estimated manually as the midway between the 2 tips along the curvilinear axis. Following this procedure, a total of two measurements is acquired per each aggregate (1 on each side). The orthogonal axis length is defined as the average between the shortest orthogonal (to the long axis) dimension and the largest one, as the sausages are not always of constant width.

'boxed' aggregates (Fig. 4): The central ROI is defined as the region between the center of the aggregate and ~1/3rd of the center-to-protrusion distance. Angles are measured with 0° being parallel to the center-to-protrusion line and 90° orthogonal to it. The neck ROI is defined as the beginning of the protrusion, where the cells are the thinnest. For later aggregates (2 and 4 dpa), the neck region is only defined if a protrusion is present, which is rare.

<u>Manual analysis of morphological features</u>

For all conditions (WT and perturbed), the morphologies of the aggregates are scored manually on regenerated, 4-5 dpa, fixed aggregates stained with phalloidin and imaged from both sides mounted between coverslips with spacers.

Heads are recognized by the presence of a +1 defect with a typical endodermal actin array circling around thinned cells. If there is no such defect but there are tentacles matching a body axis, we still score a head, as we assume that the defect is certainly hidden behind the tentacles or the edge of the sample (examples in Fig. S4B). Tentacles are thin protrusions with a well-defined ectodermal array parallel to it and visible nematocysts. We could not reliably identify feet, as their morphology is less characteristic, and we seldom observe pairs of +1/2 defects that have been associated with them (*7*). They may be present in the disordered regions that we observe in areas connecting axes. A body segment (or axis) is defined as a parallel ectodermal actin domain connecting two morphological features. Note that these morphological features are archetypes, and sometimes doubt remains about identifying the features (e.g., some have characteristics of both head and tentacle), such that our measurement is only an estimate.
Defects are counted only if directly visible, and the defects associated with tentacles are excluded. The reason is that tentacles typically do not seem to influence the actin pattern at a distance, and we want to access the defect number independently of the number of tentacles. The tentacle defects consist invariably of a +1 or 2 +1/2 at the tip (depending on its thickness) and 2 +1/2 at the basis, yielding a total charge of 0. Still, some of these defects are generally hidden by the tentacle, which would impact the analysis. We thus only scored clear non-tentacle defects, likely underestimating their number (Fig. S5B).
Body plans are scored in aggregates with more than one axis according to the diagram represented in Fig. S5D. Those are archetypal, but more variety exists in our samples (S4B).

<u>Analysis of the cell aspect ratio:</u>
The most apical planes of fixed 'boxed' aggregates were selected and maximum projected. These show the apical junctions of the cells, revealing the cell contours. The cells were then semi-manually segmented using Tissue Analyzer (*8*). The segmented images were binarized, inverted, and processed with MorpholibJ (*9*) to label the cells with connected component analysis. Regions were automatically analyzed with MorpholibJ to extract the ratio of the axes of an ellipse fit for each cell and their orientation angle.

<u>Manual analysis of Wnt intensity</u>
Image stacks are stitched using the Zen software then a maximum intensity projection is performed. The HCR *in situ* signal is characterized by numerous, small, sub-resolution bright dots, as shown in Fig. S8C,D,E. This signal is specific, as performing an HCR with the same detection and amplification protocol but with a probe targeting an absent mRNA (here GFP) presents some background signal, but not the bright sub-resolution dots (Fig. S8G). To remove part of the background signal, the *Wnt* maximum projection images are processed using a 20-pixel (for measurements) or 50-pixel (for display) rolling ball background subtraction (using Fiji). The ROI for the protrusion tip and neck are defined on the actin channel, with the tip being the region with rounded cells and the neck with elongated actin fibers. The average intensity is then measured using the same ROIs on the *Wnt* channel with the Fiji measurement tool. For comparison, two large ROIs are defined elsewhere in the actin channel, and their respective average actin intensity is measured on the *Wnt* channel. The maximum *Wnt* intensity ROI is chosen manually on the *Wnt* channel to be the region with seemingly the most actin and a diameter similar to that of the neck ROI. The minimum *Wnt* intensity ROI is defined similarly but on the seemingly minimum signal with a diameter similar to the protrusion region. All ROIs are chosen to avoid the stitching area artifact (cross-shaped lower-intensity regions generated by the image stitching process).

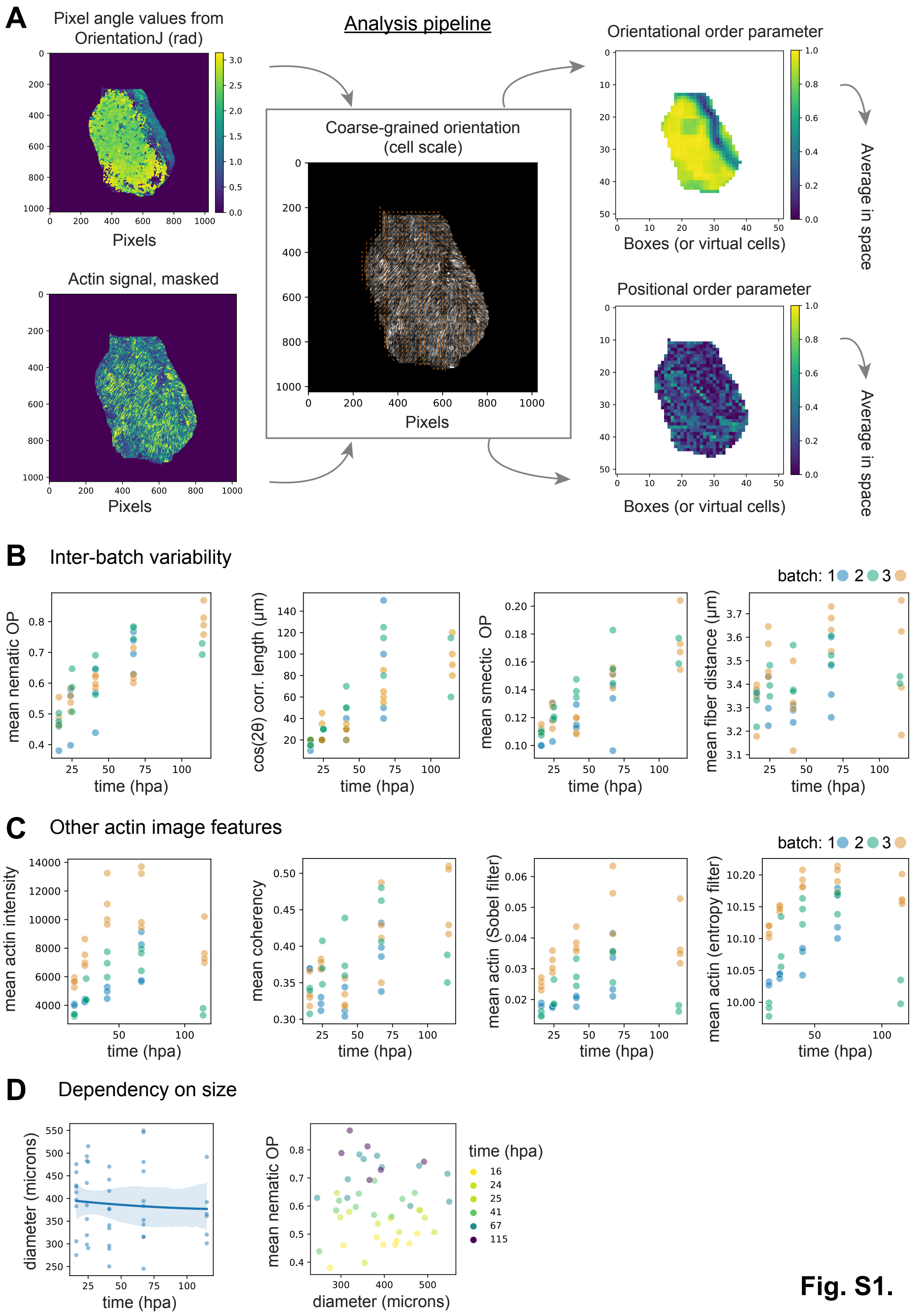

A
Pixel angle values from OrientationJ (rad)
Pixels
Actin signal, masked
Pixels
Analysis pipeline
Coarse-grained orientation (cell scale)
Pixels
Orientational order parameter
Boxes (or virtual cells)
Average in space
Positional order parameter
Boxes (or virtual cells)
Average in space
B
Inter-batch variability
batch: 1 2 3
mean nematic OP
cos(2θ) corr. length (µm)
mean smectic OP
mean fiber distance (µm)
time (hpa)
C
Other actin image features
batch: 1 2 3
mean actin intensity
mean coherency
mean actin (Sobel filter)
mean actin (entropy filter)
time (hpa)
D
Dependency on size
diameter (microns)
time (hpa)
mean nematic OP
diameter (microns)
time (hpa)
16
24
25
41
67
115

Fig. S1.

Fig. S1.

(**A**) Analysis pipeline principle. A map of the angle's values at the fiber (i.e. 2-pixels) scale is obtained with OrientationJ. This map and a mask of the sample based on the actin signal are combined and coarse-grained at the cellular scale (central image). From this, the orientational and positional order parameter maps are obtained, and then averaged spatially. (**B**) The same plots as **Fig. 1C** and **D**, but with the batch of each aggregate color-coded. (**C**) Actin mean intensity, mean coherency, mean contrast (as measured with a Sobel filter and with an entropy filter), as a function of time. These measurements show that the order parameter evolution is not trivially dependent on image intensity or contrast. (**D**) Left, aggregate diameter with time. Right, nematic order parameter as a function of the aggregate diameter color-coded for different times. The line is a 2nd-order polynomial regression as a visual guide, with a lighter area representing the confidence intervals. (**B-D**) One dot is one aggregate.

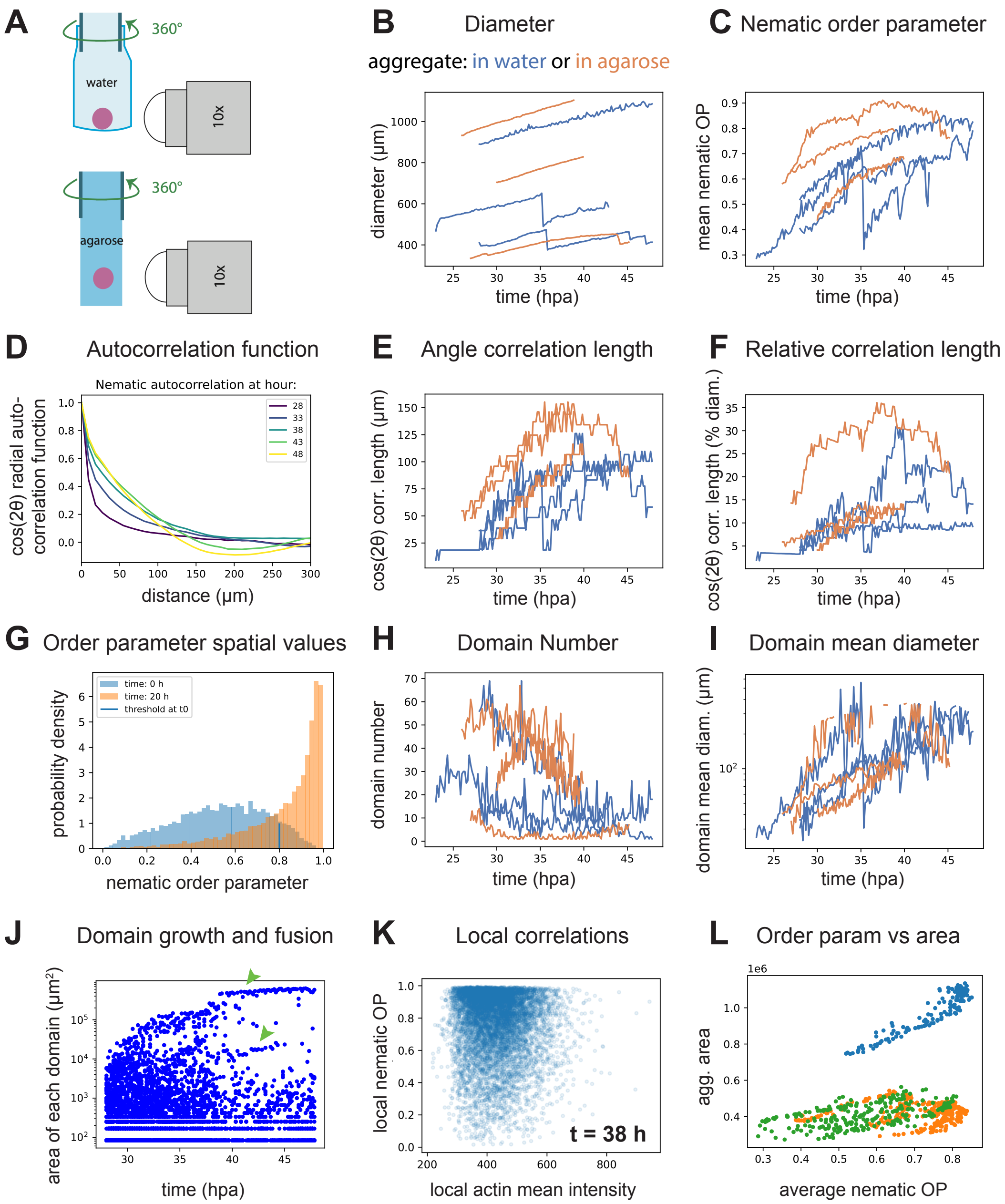
A
360°
water
10x
360°
agarose
10x
B
Diameter
aggregate: in water or in agarose
diameter (µm)
time (hpa)
C
Nematic order parameter
mean nematic OP
time (hpa)
D
Autocorrelation function
Nematic autocorrelation at hour:
28
33
38
43
48
cos(2θ) radial auto-correlation function
distance (µm)
E
Angle correlation length
cos(2θ) corr. length (µm)
time (hpa)
F
Relative correlation length
cos(2θ) corr. length (% diam.)
time (hpa)
G
Order parameter spatial values
time: 0 h
time: 20 h
threshold at t0
probability density
nematic order parameter
H
Domain Number
domain number
time (hpa)
I
Domain mean diameter
domain mean diam. (µm)
time (hpa)
J
Domain growth and fusion
area of each domain (µm²)
time (hpa)
K
Local correlations
local nematic OP
t = 38 h
local actin mean intensity
L
Order param vs area
1e6
agg. area
average nematic OP

Fig. S2.

Fig. S2.

(**A**) Schematic of light-sheet sample mounting in water or agarose. The pink disks are aggregates, the green lines indicate glass capillary sections, and on the right is the detection objective. (**B**) Time evolution of the diameters of 3 live LifeAct-GFP aggregates in water (blue lines) and 3 in agarose (orange lines) from N=6 batches. (**C**) Spatial average of the orientational order parameter (nematic OP) with time of the same samples. (**D**) Autocorrelation function of cos(2θ) used to determine the correlation length in **Fig. 2D**, at 5 time-points (in hpa) from the aggregate in **Fig. 2A**. (**E**) Correlation length (defined as in **Fig. 1C**) with time of the same 6 samples. (**F**) Correlation length normalized by the sample's diameter at each time point. (**G**) Distribution of the spatial nematic order parameter values of the aggregate in **Fig. 2A**, with the 10% threshold used in the definition of the high-order domains (t0 is 28 hpa). After 20 h, many more domains have reached the threshold. (**H**) High-order domains number with time of the same 6 samples. (**I**) Mean high-order domain diameters with time of the same 6 samples. (**J**) Individual areas of high-order domains with time in the aggregate in **Fig. 2A**. One dot is one domain at one time point. The arrows point at two identified domain trajectories, one of a big and one of a medium domain. (**K**) Local correlation (or absence thereof) between actin intensity and order parameter at 38 hpa. One dot is one box (spatial area). (**L**) Aggregate area vs nematic order parameter (OP) for the three aggregates of **Fig. 2**, each in one color.

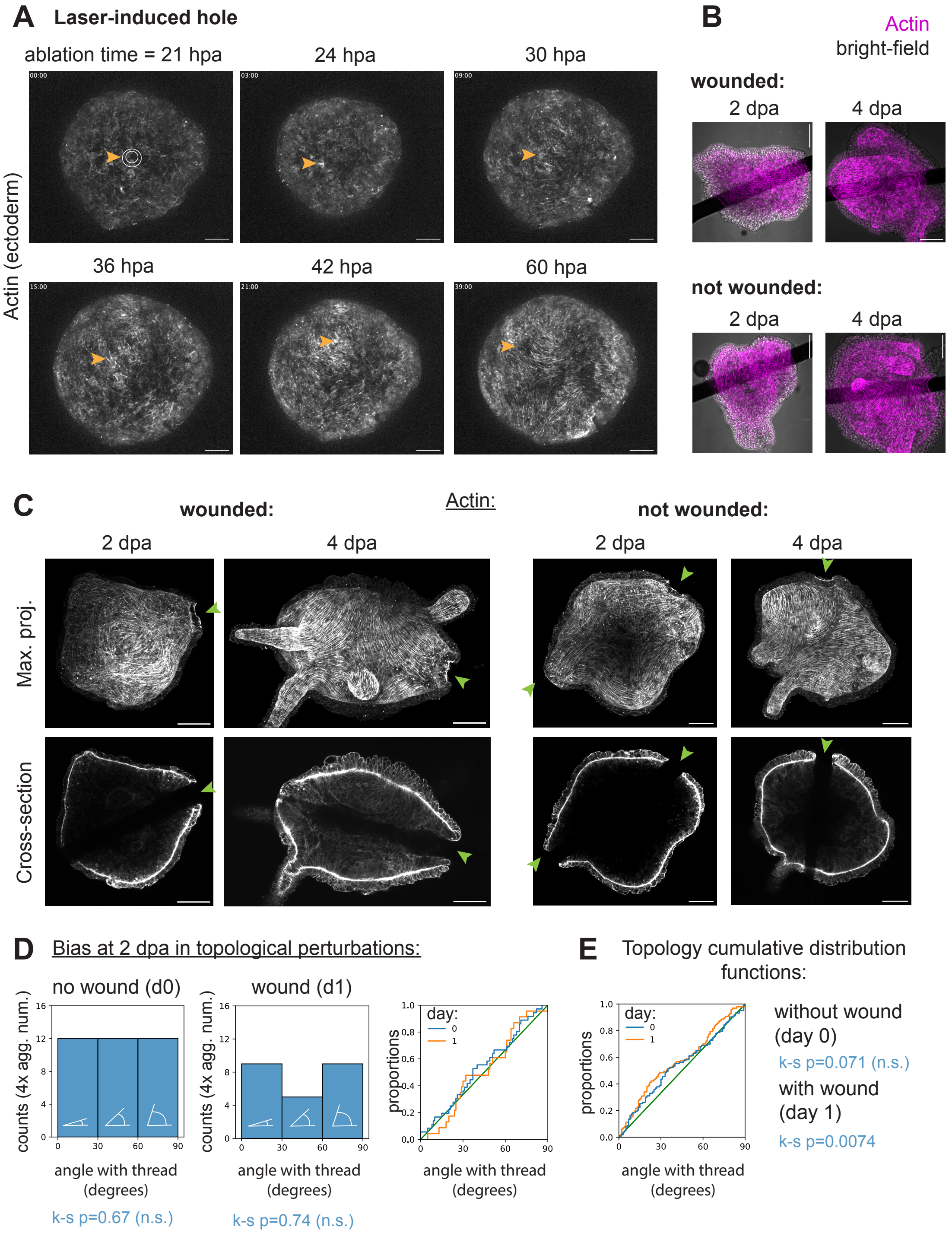

Fig. S3: topology

Fig. S3: topology.
(**A**) Six time points of a spinning-disk microscopy movie of actin in the ectoderm (LifeAct-GFP) of an aggregate in agarose punctured with a laser at t=21 hpa. The orange arrows track the area of the puncture, circled in white on the first image (inner circle: initial puncture, outer circle: recoil). (**B**) Composite images of the bright field channel (black and white) and actin (magenta) of the aggregates in **Fig. 3A**,**B**. (**C**) Maximum projections and single plane cross-sections of phalloidin stainings of aggregates pierced by a thread either at 1 dpa (wounded, left) or at 0 dpa (not wounded, right). The green arrows show the holes in the epithelia made by the nylon thread. Representative images of the aggregates measured in **Fig. 3** (**D**) Similar measurements as in **Fig. 3B,D** but at 2 dpa. n=7 d1 aggregates and n=9 d0 aggregates from N=1 batch each. (**E**) Cumulative distribution functions (cdf) of all measurements used to produce the histograms in **Fig. 3B** (orange line) and **3D** (blue line), and of the cdf of a uniform distribution (green line). All microscopy images are maximum-intensity stack projections. All scalebars are 100 microns.

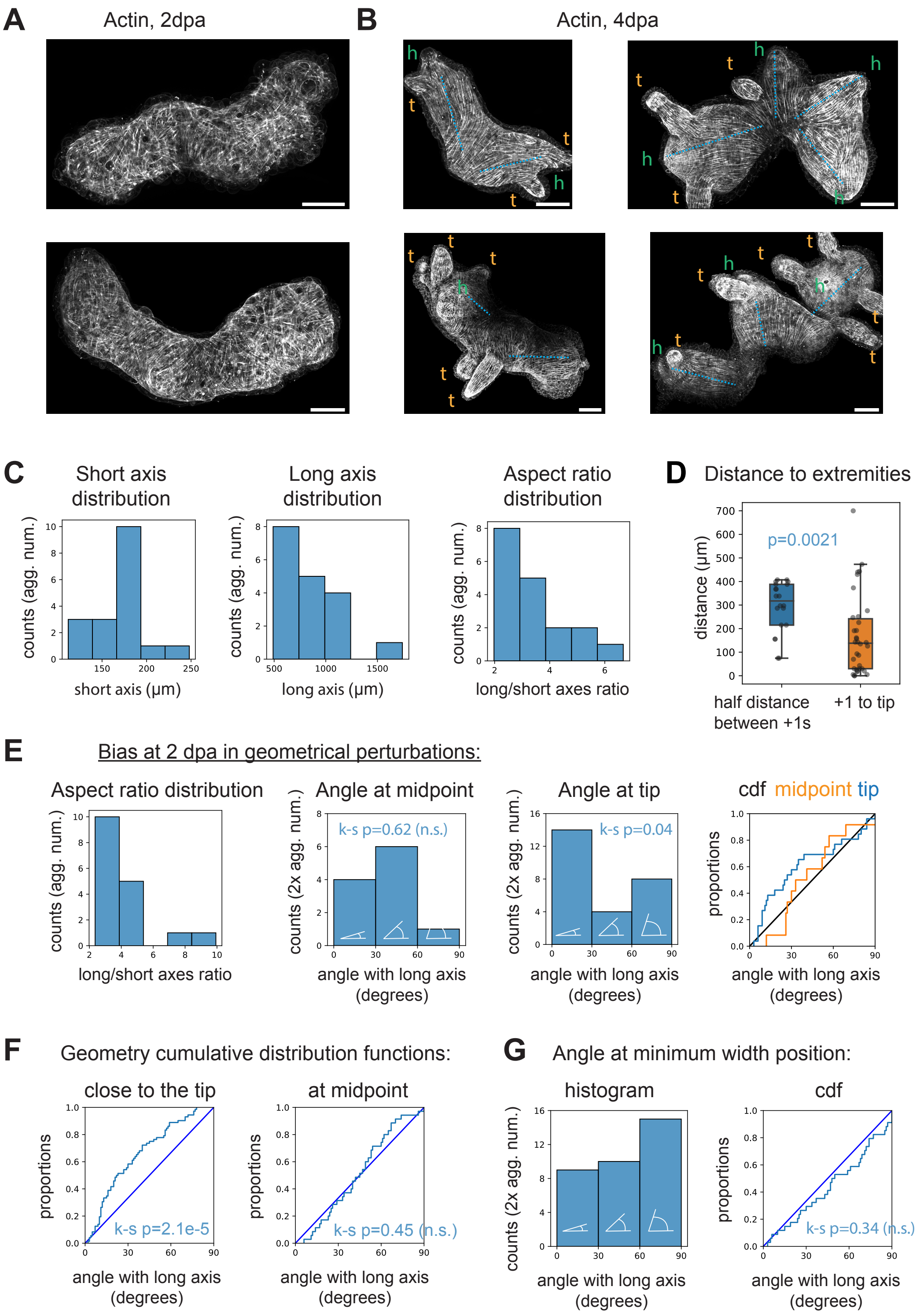

Fig. S4: geometry

Fig. S4: geometry.

(**A**) Additional examples of 'sausages' at 2 dpa as in **Fig. 3E**. (**B**) Additional examples of 'sausages' at 4 dpa as in **Fig. 3E**. Heads are marked with an 'h', tentacles with a 't', and body segments with a dashed line. (**C**) Histograms of the short and long axes of the samples analyzed and of their aspect ratio. (**D**) Boxplot of half distance between +1 defects (left) and of distance between +1 and tip (right). One dot is one measurement point. (**E**) similar measurements as **Fig. 3H** and **Fig. S4A,B** but at 2 dpa. n=7 aggregates from N=1 batch (**F**) Cumulative distribution functions (cdf) of all measurements used to produce the histograms in **Fig. 3H** and of the cdf of a uniform distribution (blue line) (**G**) Manual measurements of the angle of actin with respect to the long axis at the position of minimum width along the same axis. Left: histogram, right: cdf. All microscopy images are maximum-intensity stack projections. All scalebars are 100 microns.

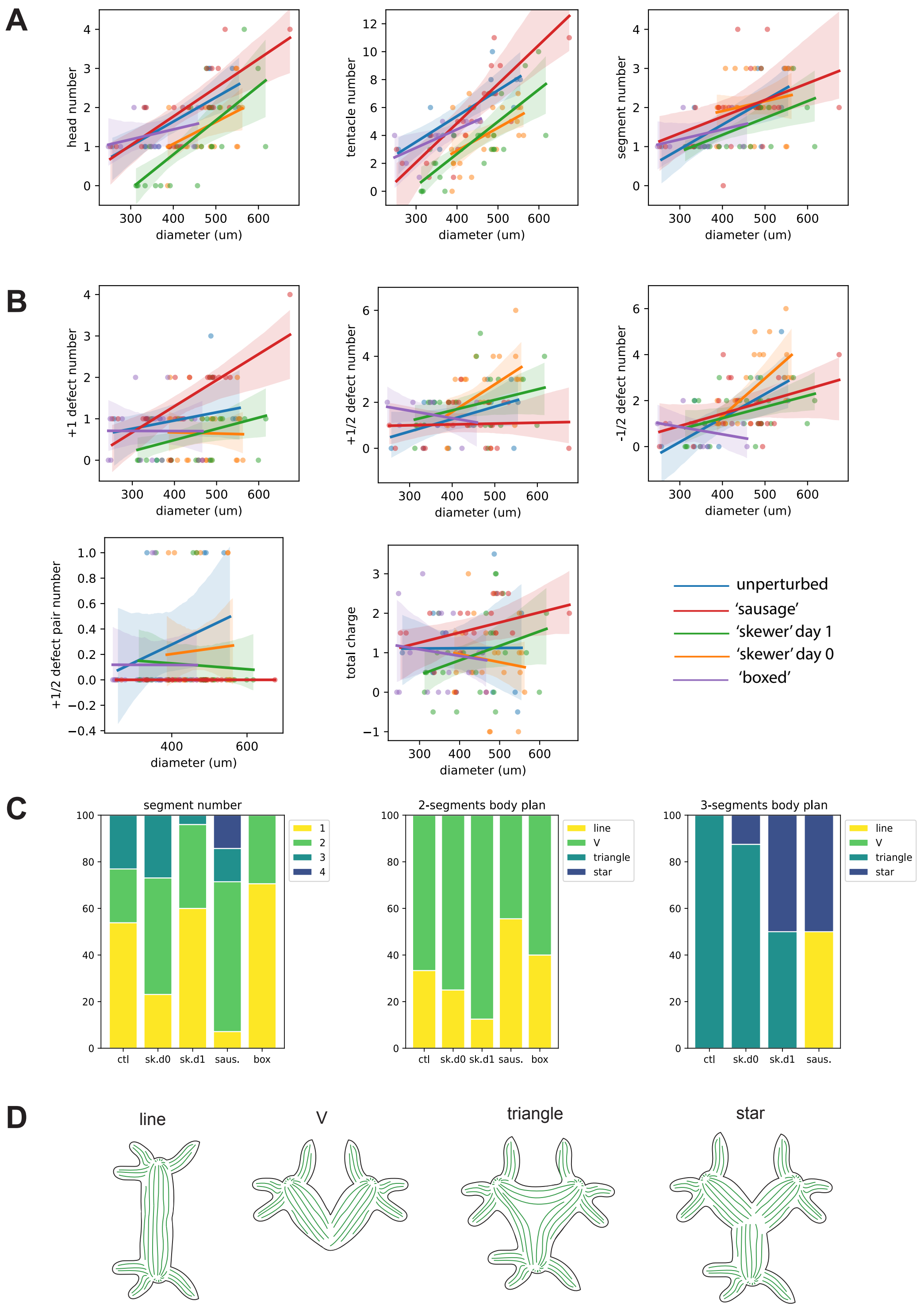

Fig. S5: morphologies

Fig. S5: morphologies.

(**A**) Head, tentacle, and segment numbers as a function of aggregate equivalent diameter for all the perturbations of **Fig. 3** and **4**. (**B**) Different types of defects and total defect charge as a function of aggregate equivalent diameter for all the perturbations of **Fig. 3** and **4**. (**A**) and (**B**): each dot is an aggregate, the lines are 1st-order polynomial regression as a visual guide, with a lighter area representing the confidence intervals. (**C**) Barplots of segment number and body plan distribution (in %) across perturbations. 2-segment and 3-segment body plans are visualized separately as their proportions differ between perturbations. (**D**) Schematic of the body plan classification in (**C**). For all measurements, unperturbed: n=13 aggregates from N=2 batches, ‘sausages’: n=20, N=3, ‘skewers’ d0: n=26, N=3, ‘skewers’ d1: n=25, N=3, ‘boxed’: n=9, N=1.

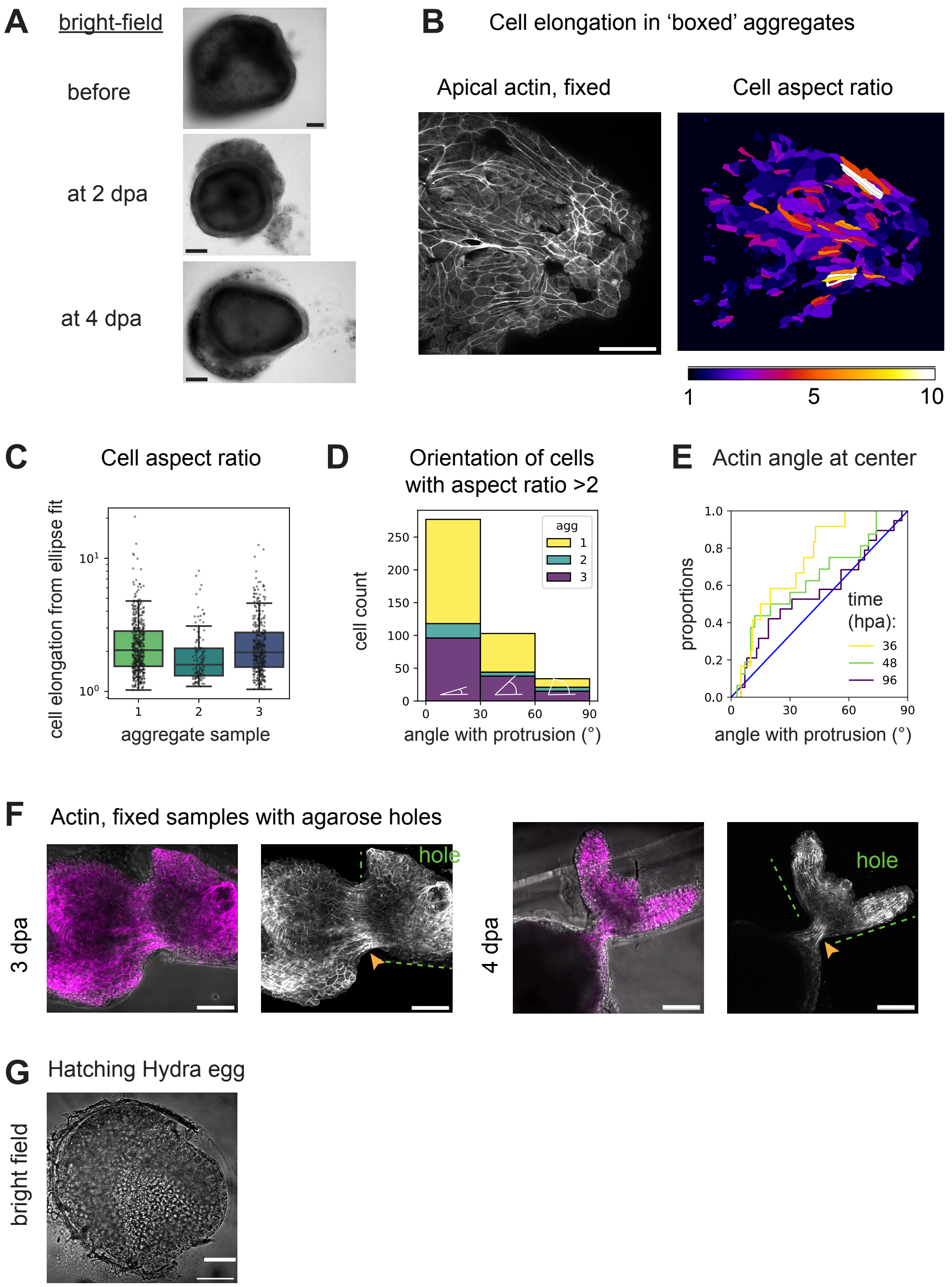
A
bright-field
before
at 2 dpa
at 4 dpa
B
Cell elongation in 'boxed' aggregates
Apical actin, fixed
Cell aspect ratio
1
5
10
C
Cell aspect ratio
cell elongation from ellipse fit
aggregate sample
D
Orientation of cells with aspect ratio >2
cell count
agg
angle with protrusion (°)
E
Actin angle at center
proportions
time (hpa):
36
48
96
angle with protrusion (°)
F
Actin, fixed samples with agarose holes
3 dpa
4 dpa
hole
hole
G
Hatching Hydra egg
bright field

Fig. S6.

Fig. S6.

(**A**) Bright-field images of the aggregates shown in **Fig. 4B-D**. (**B**) Left, max. projection of the four most apical planes of protrusion #1 shown in **Fig. 4G**, showing the cells' apical junctions. Right, cells of the same image, segmented and color-coded with their aspect ratio. Dark blue is an aspect ratio of 1 (round cells), orange is an aspect ratio of 5, and white is an aspect ratio superior to 10. (**C**) Box plot of cell aspect ratios in 3 different samples. One dot is one cell. (**D**) Histogram of the orientation of the cells' longest axis compared to the protrusion angle. Each sample is color-coded and stacked on top of the other samples. (**E**) Cumulative distribution function of the measurements used to produce the histograms in **Fig. 4E**. (**F**) Maximum projections of microscopy images of actin (phalloidin) in WT aggregates placed in agarose boxes with holes (agarose edges in green dashed lines) at 1 dpa and fixed at 3-4 dpa. Both examples show a striking alignment of actin at the neck (orange arrows). (**G)**. Bright-field image of the embryo in **Fig. 4H**. All scalebars are 100 microns.

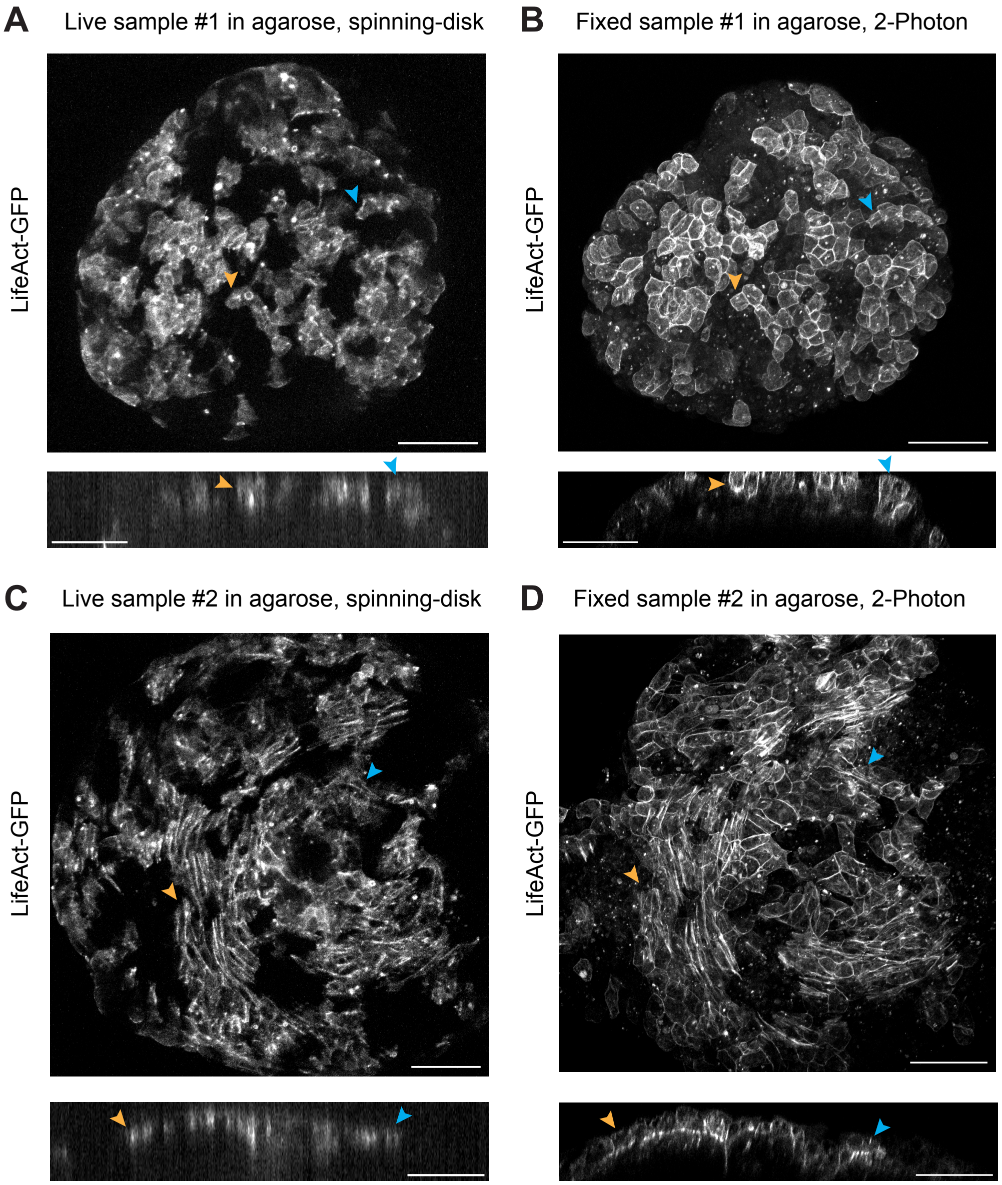
A
Live sample #1 in agarose, spinning-disk
B
Fixed sample #1 in agarose, 2-Photon
LifeAct-GFP
LifeAct-GFP
C
Live sample #2 in agarose, spinning-disk
D
Fixed sample #2 in agarose, 2-Photon
LifeAct-GFP
LifeAct-GFP

Fig. S7

Fig. S7.

(**A**) Maximum projection and side view of a live, 1 dpa, mosaic aggregate embedded in agarose. LifeAct-GFP is imaged with a spinning disk microscope. This sample does not show actin fibers yet. (**B**) Same sample, fixed immediately after live imaging, and imaged with 2-Photon microscopy. The mosaic allows recognizing individual cells (orange and blue arrows) and shows that **A** faithfully represents the basal actin fibers, without detecting much unwanted signal from the cell junctions. (**C**) Aggregate from the same batch as **A** and imaged similarly, but with already developed actin fibers. This is normal batch variability of fiber development, and these two examples represent two extremes of a batch of 6 aggregates. (**D**) The fibers are also faithfully detected when compared to 2-Photon microscopy. All scalebars are 100 microns.

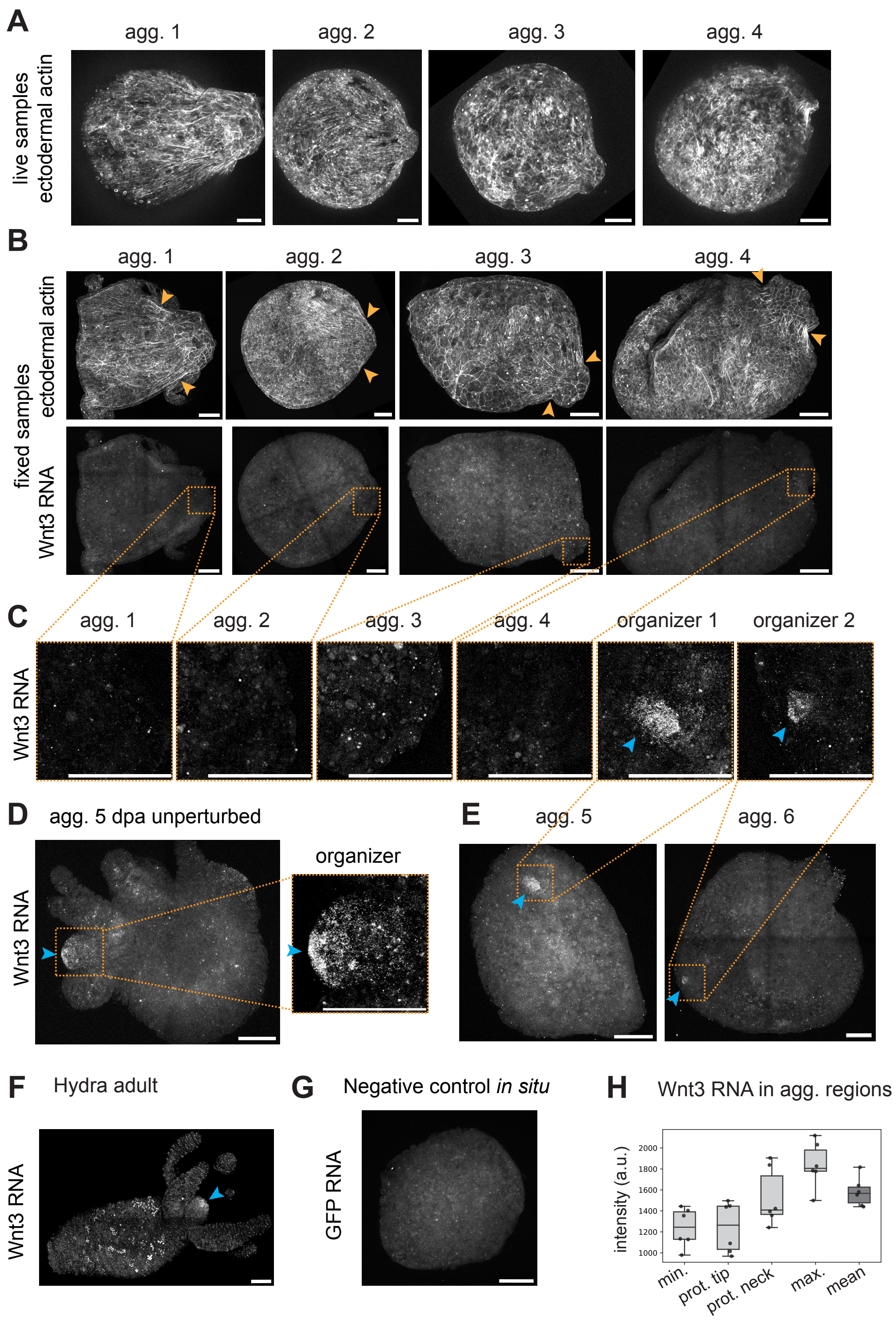
A
agg. 1
agg. 2
agg. 3
agg. 4
live samples
ectodermal actin
B
fixed samples
ectodermal actin
Wnt3 RNA
C
organizer 1
organizer 2
Wnt3 RNA
D
agg. 5 dpa unperturbed
organizer
E
agg. 5
agg. 6
F
Hydra adult
G
Negative control *in situ*
GFP RNA
H
Wnt3 RNA in agg. regions
intensity (a.u.)
2000
1800
1600
1400
1200
1000
min.
prot. tip
prot. neck
max.
mean

Fig. S8

Fig. S8.

(**A**) Live microscopy images of actin (LifeAct-GFP) in 4 of the aggregates of **Fig. 4G** before fixation. (**B**) Large-field images of these 4 aggregates. Top: actin (LifeAct-GFP). Orange arrows: neck sides. The protrusions can be identified with those in **A**. Bottom: raw *Wnt3* RNA signal. (**C**) Close-ups of the images in **B** and **E**, with additional background subtraction using a rolling ball algorithm. The blue arrows show well-defined head organizers with numerous sub-resolution bright dots. The contrast and scale is set identically in all the close-ups. (**D**) Left, *Wnt3* RNA in a regenerated 5 dpa aggregate. One head organizer is visible (blue arrow). Right, close-up with background subtraction on the most apparent head organizer. (**E**) Aggregates from the same batch as **B** that show Wnt3 head organizers. Contrast is set similarly on all the aggregates of **B** and **E**. (**F**) HCR RNA *in situ* detection of *Wnt3* on an adult Hydra. Blue arrow: head organizer. The bright cells in the body and tentacles are autofluorescent nematoblasts. (**G**) Control HCR *in situ* performed using antisense probes specific for GFP mRNA, not expressed by the WT Hydra. Representative of 3 aggregates. (**H**) Boxplots of **Fig. 4H** before the normalization by the mean aggregate signal. All fluorescence microscopy images are maximum-intensity stack projections. All scalebars are 100 microns.

Movie S1 (separate file). Example of a WT aggregate regeneration in water over days, bright-field imaging. Scalebar: 100 microns. Timestamps: hours after aggregation (hpa).

Movie S2 (separate file). Actin-tagged (LifeAct-GFP) aggregate regeneration in water from 28 hpa to 48 hpa, mounted in an FEP cuvette and imaged with a light-sheet microscope. Scalebar: 100 microns. Timestamps: hours after aggregation (hpa).

Movie S3 (separate file). Nematic order parameter value calculated from the aggregate in Movie S2, superimposed on the actin Movie S2. X and Y axis: position in pixels. Colormap: nematic order parameter value. Timestamps: hours after aggregation (hpa).

Movie S4 (separate file). Zoomed-in view of a fusion event in Actin-tagged (LifeAct-GFP) aggregate regeneration in agarose. The orange arrows show two domains with orthogonal orientation initially that later end up parallel to each other. Scalebar: 10 microns. Timestamps: hours after aggregation (hpa).

Movie S5 (separate file). LifeAct-GFP aggregate regeneration after laser puncture at 21 hpa, mounted in 1% agarose and imaged with a spinning-disk microscope. Scalebar: 100 microns. Scalebar: 100 microns. Timestamps: hours after aggregation (hpa).

Movie S6 (separate file). Bright-field movie of the regeneration of an aggregate pierced by a thread at 1 dpa, free-floating in water. Scalebar: 500 microns. Timestamps: hours after aggregation (hpa).

Movie S7 (separate file). Full z-stack of a phalloidin staining of a 4 dpa aggregate pierced by a thread at 1 dpa, labeled with depth in microns. Scalebar: 100 microns.

Movie S8 (separate file). Bright-field movie of the regeneration of a ‘sausage’ aggregate, free-floating in water. Scalebar: 500 microns. Timestamps: hours after aggregation (hpa).

Movie S9 (separate file). Full z-stack of a phalloidin staining of a 4 dpa ‘sausage’ aggregate, labeled with depth in microns. Scalebar: 100 microns.

Movie S10 (separate file). LifeAct-GFP aggregate regeneration in a 2% agarose box with a hole, imaged just after hole creation, from 22 hpa to 45 hpa, with a spinning-disk microscope. Scalebar: 100 microns. Timestamps: hours after aggregation (hpa).

Movie S11 (separate file). Later LifeAct-GFP aggregates regeneration in an agarose box with a hole (after being imaged as in Movie S5) from 45 hpa to 88 hpa, bright-field imaging. Scalebar: 100 microns. Timestamps: hours after aggregation (hpa). The aggregates can move inside the agarose (left aggregate) or stay relatively still (right aggregate).

Movie S12 (separate file). Aggregate inflation in agarose, with cells labeled with a lipid dye. Scalebar: 100 microns. Timestamps: hours after aggregation.

1. A. Seybold, W. Salvenmoser, B. Hobmayer, Sequential development of apical-basal and planar polarities in aggregating epitheliomuscular cells of Hydra. *Developmental Biology* **412**, 148–159 (2016).

2. R. Aufschnaiter, R. Wedlich-Söldner, X. Zhang, B. Hobmayer, Apical and basal epitheliomuscular F-actin dynamics during *Hydra* bud evagination. *Biology Open*, bio.022723 (2017).

3. H. M. T. Choi, M. Schwarzkopf, M. E. Fornace, A. Acharya, G. Artavanis, J. Stegmaier, A. Cunha, N. A. Pierce, Third-generation in situ hybridization chain reaction: multiplexed, quantitative, sensitive, versatile, robust. *Development* **145**, dev165753 (2018).

4. B. C. Vellutini, M. B. Cuenca, A. Krishna, A. Szałapak, C. D. Modes, P. Tomančák, Patterned embryonic invagination evolved in response to mechanical instability. bioRxiv [Preprint] (2023). https://doi.org/10.1101/2023.03.30.534554.

5. E. Kuehn, D. S. Clausen, R. W. Null, B. M. Metzger, A. D. Willis, B. D. Özpolat, Segment number threshold determines juvenile onset of germline cluster expansion in Platynereis dumerilii. *Journal of Experimental Zoology Part B: Molecular and Developmental Evolution* **338**, 225–240 (2022).

6. Z. Püspöki, M. Storath, D. Sage, M. Unser, "Transforms and Operators for Directional Bioimage Analysis: A Survey" in *Focus on Bio-Image Informatics*, W. H. De Vos, S. Munck, J.-P. Timmermans, Eds. (Springer International Publishing, Cham, 2016; https://doi.org/10.1007/978-3-319-28549-8_3), pp. 69–93.

7. Y. Maroudas-Sacks, L. Garion, L. Shani-Zerbib, A. Livshits, E. Braun, K. Keren, Topological defects in the nematic order of actin fibres as organization centres of Hydra morphogenesis. *Nat. Phys.* **17**, 251–259 (2021).

8. B. Aigouy, D. Umetsu, S. Eaton, "Segmentation and Quantitative Analysis of Epithelial Tissues" in *Drosophila: Methods and Protocols*, C. Dahmann, Ed. (Springer, New York, NY, 2016; https://doi.org/10.1007/978-1-4939-6371-3_13), pp. 227–239.

9. D. Legland, I. Arganda-Carreras, P. Andrey, MorphoLibJ: integrated library and plugins for mathematical morphology with ImageJ. *Bioinformatics* **32**, 3532–3534 (2016).
